# Isolated increased free-triiodothyronine in medication-naïve children and adolescents with ADHD: a retrospective observational study

**DOI:** 10.3389/fendo.2026.1915311

**Published:** 2026-10-09

**Authors:** Hervé Caci, Philippe Caron

**Affiliations:** 1 Service Universitaire de Pédiatrie, Hôpitaux Pédiatriques de Nice CHU-Lenval, Nice, France; 2 Service d’Endocrinologie, maladies métaboliques et nutrition, Centre Hospitalier Universitaire de Toulouse, Toulouse, France

**Keywords:** ADHD, biomarker, children and adolescents, deiodinases, free triiodothyronine, pediatric reference intervals, puberty, thyroid hormones

## Abstract

**Background:**

Thyroid hormones, free triiodothyronine (free-T3) in particular, are crucial for fetal development and child growth and may influence neurodevelopmental disorders including Attention-Deficit/Hyperactivity Disorder (ADHD) in children and adolescents. Yet data are scarce and inconsistent, often relying on adult rather than pediatric reference intervals or on incomplete thyroid panels, and the value of free-T3 as a biomarker across development remains unclear.

**Methods:**

In this retrospective observational study, we characterized thyroid function in medication-naïve patients aged 6 to 17 years with ADHD, using community laboratory results collected between March 2004 and December 2022 in a university pediatric department. Thyroid testing was prescribed prior to methylphenidate initiation. We determined the percentage of patients with free-T3, free-T4, and TSH levels below, within, or above age- and sex-adjusted reference intervals. In the largest immunoassay subgroup (ADVIA Centaur; n = 404), individual free-T3 values were converted to z-scores using published pediatric normative data. A longitudinal ancillary analysis assessed temporal stability in patients with repeat measurements. No non-ADHD comparison group was available.

**Results:**

Among 715 medication-naïve patients with ADHD (570 boys, 145 girls) with normal TSH, approximately one-third presented with isolated increased free-T3 concentrations (2IfT3 profile) — 33.68% of boys, 27.59% of girls — the most frequent thyroid anomaly observed. In the ADVIA Centaur subgroup, free-T3 z-scores were higher than the pediatric norms across all age groups, with the largest deviation in boys aged 11–14 years. Among 57 patients with repeat testing (median interval 2.57 years), instability was marked: 53.57% of those initially showing 2IfT3 normalized, while 50% of those initially normal developed 2IfT3.

**Conclusion:**

Isolated increased free-T3 is frequent in medication-naïve pediatric ADHD and most pronounced in boys aged 11–14 years, but its marked temporal instability precludes its use as a stable biomarker. In the absence of a concurrent non-ADHD comparison group, these findings describe the frequency of this biochemical profile within an ADHD cohort rather than establishing an association with ADHD itself. The 2IfT3 profile may reflect a developmentally and state-dependent neuroendocrine pattern, and warrants prospective study incorporating direct pubertal staging, standardized assay methodology, and appropriate controls.

## 1 Introduction

The thyroid gland secretes thyroxine (T4) and, in smaller amounts, triiodothyronine (T3). Most circulating T3 derives from outer-ring deiodination of T4 in peripheral tissues, catalyzed by the selenium-dependent type 1 and type 2 deiodinases (DIO1, DIO2), whereas type 3 deiodinase (DIO3) inactivates thyroid hormones through inner-ring deiodination, producing reverse-T3 from T4 and T2 from T3 (1). Cellular uptake requires specific transmembrane transporters, chiefly monocarboxylate transporter 8 (MCT8) and MCT10, with OATP1C1 mediating brain-specific transport (2). Free-T3, the biologically active form, regulates gene expression through the nuclear thyroid hormone receptors TRα and TRβ. Circulating free-T3 and free-T4 are maintained within narrow limits by hypothalamic–pituitary feedback involving thyrotropin-releasing hormone (TRH) and thyroid-stimulating hormone (TSH). Biochemically, thyrotoxicosis is characterized by increased free-T3 and free-T4 with suppressed TSH, whereas primary hypothyroidism presents with low free-T3 and free-T4 and increased TSH.

Thyroid hormones are essential to brain development from conception through late childhood (3). Free-T3 and free-T4 concentrations are considerably higher in infancy than in adulthood, with wide reference intervals reflecting substantial interindividual variability (4–6). Growth hormone enhances peripheral T4-to-T3 conversion through increased DIO1 activity, particularly during growth spurts. Sex differences emerge during adolescence: prepubertal free-T3 elevation in males normalizes by adulthood (6), and a TSH dimorphism appears in adulthood, women showing levels 0.27 mIU/L higher than men (7). These physiological variations require age-, sex-, and ideally puberty-specific reference intervals (RIs) for accurate interpretation in pediatric populations (8). RIs also vary appreciably across analytical methods, assay manufacturers, and source populations, which complicates comparison between studies.

Beyond neurodevelopment and metabolic regulation, thyroid hormones have been implicated in psychiatric conditions in the absence of overt thyroid diseases (9). Increased T3 has been reported repeatedly in post-traumatic stress disorder (PTSD), in combat veterans (10–16) and in women with trauma histories (17, 18), with positive correlations to hyperarousal severity (16) and to the Novelty Seeking dimension (15) (**Supplementary Table 1**). A parallel literature, predominantly from Swedish cohorts, has linked increased total-T3 and an increased T3/T4 ratio to delinquency (19, 20), criminal recidivism (21–23), and psychopathy scores (24), with some studies noting concurrent childhood attention difficulties (25). Together, these observations raise the question of whether a comparable pattern of increased peripheral T3 occurs in Attention-Deficit/Hyperactivity Disorder (ADHD), which shares clinical features and comorbidity patterns with these conditions (**Figure 1**).

**Figure 1:**
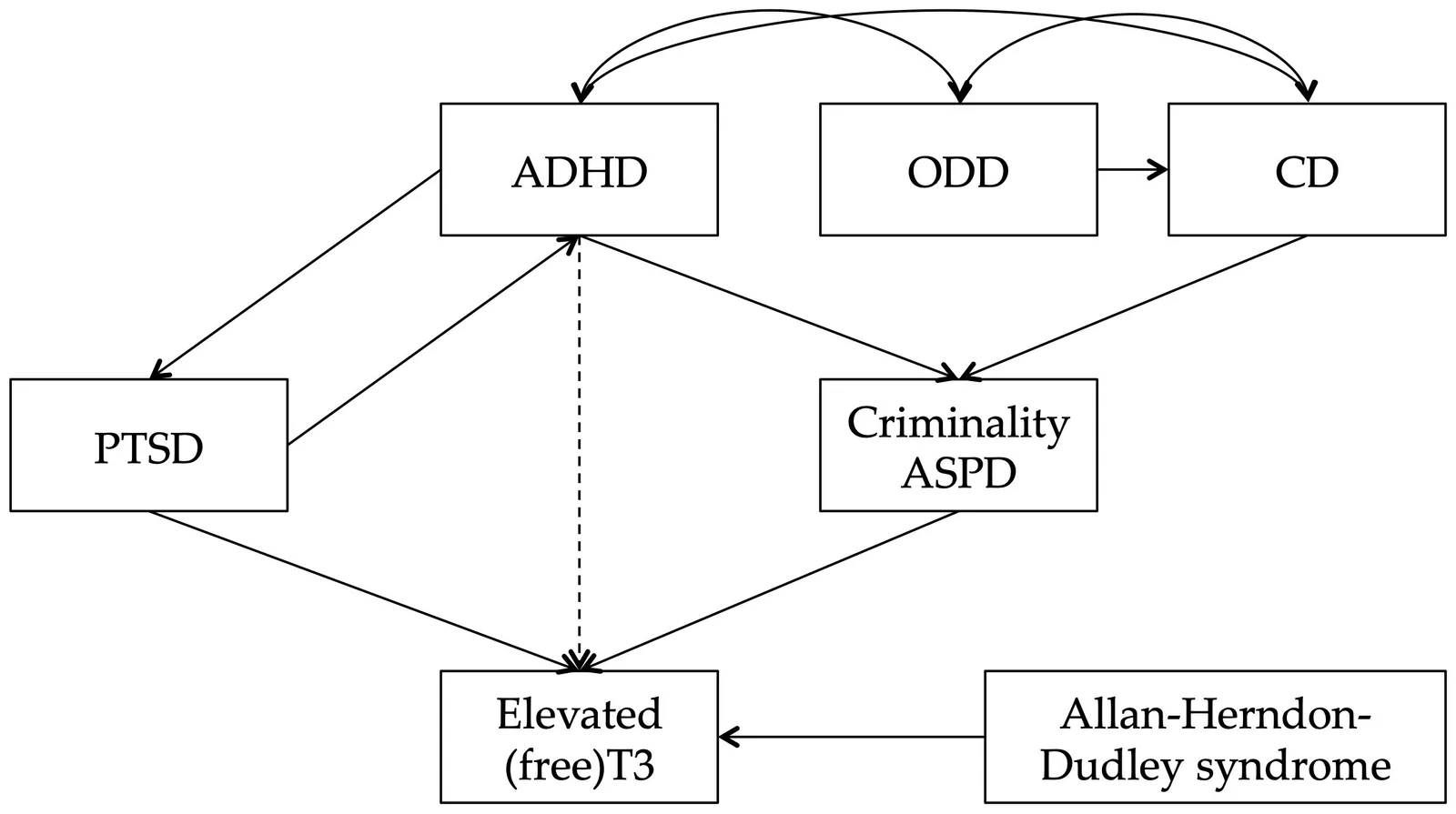
Conceptual framework linking ADHD to thyroid hormone alterations through shared comorbidities and developmental pathways. Solid arrows represent established clinical associations and developmental pathways (ADHD comorbidities: ODD, CD, PTSD, criminality/ASPD). PTSD and antisocial behaviors associate with increased free-T3 (solid arrows). The dashed arrow represents the research question: does ADHD associate with altered thyroid profiles? Allan-Herndon-Dudley syndrome (MCT8 mutations) provides a genetic model of isolated T3 increase with neurodevelopmental impairment.

ADHD is a neurodevelopmental disorder characterized by developmentally inappropriate levels of inattention, hyperactivity, and impulsivity (26), whose expression fluctuates across the lifespan under developmental and environmental influences (27, 28). Several lines of evidence connect it to thyroid function. Clinically, cognitive and motor hyperactivity overlap with features of thyrotoxicosis and of resistance to thyroid hormone β (RTHβ), an autosomal dominant condition caused by THRB mutations in which ADHD-like presentations have been described (29–32). Resistance to thyroid hormone receptor α shares part of this phenotypic territory, with high-normal T3, low-normal T4, subnormal reverse-T3, and normal TSH (33–35). Epidemiologically, ADHD is associated with autoimmune thyroid disease in first-degree relatives and with increased risks of both hypothyroidism and thyrotoxicosis, although absolute rates remain low (36, 37); maternal thyroid autoimmunity during pregnancy has been associated with increased offspring ADHD risk (38).

Studies measuring thyroid hormones directly in ADHD are heterogeneous (**Supplementary Table 2**). Increased total-T3 levels have been reported in 14–37% of patients in small samples, generally with normal TSH and T4 (39–41), and TSH concentrations were normal in 98.50% to 99.43% of patients across cohorts (40, 42–44). A 2025 meta-analysis of 54 studies found significantly higher free-T3 in children and adolescents with ADHD than in controls (SMD = 0.43; 95% CI [0.12; 0.75]; p = 0.007), with TSH unremarkable across subgroups (45). These studies were nonetheless limited by heterogeneous diagnostic criteria, small samples, incomplete thyroid panels, and inconsistent or unreported reference intervals. Critically, none accounted for immunoassay platform heterogeneity, and none applied age- and sex-specific reference intervals to individual values. A higher group mean says little about how many individual patients exceed their own reference range, or by how much.

This gap defines the present study. Using a single-center clinical database accumulated over nineteen years, we characterized thyroid function in medication-naïve children and adolescents with ADHD by classifying each individual free-T3, free-T4, and TSH values against age- and sex-specific reference intervals rather than pooled adult cut-offs, and by adjusting for immunoassay platform. Our primary aim was to describe the frequency, within this cohort, of an isolated increase in free-T3 with normal free-T4 and TSH (hereafter the 2IfT3 profile), and how that frequency varies with age and sex. A secondary aim, using repeated measurements available in a subset of patients, was to assess whether the profile is stable over time — a prerequisite for any use as a biomarker. No non-ADHD comparison group was available; the study is therefore descriptive and hypothesis-generating.

## 2 Materials and methods

### 2.1 Study design and setting

This retrospective observational study was conducted at a French university hospital outpatient unit from March 2004 to December 2022. Children and adolescents aged 6–17 years were referred for assessment when parents reported attention difficulties and/or excessive motor activity. The first author performed all ADHD diagnoses according to DSM-IV or DSM-5 criteria through comprehensive face-to-face clinical interviews with patients and their parents, typically lasting two hours, and was blind to thyroid results at the time of diagnosis. Longitudinal data were available in a subset of patients who underwent a second thyroid panel; these analyses are ancillary to the primary cross-sectional design. This study is reported in accordance with the Strengthening the Reporting of Observational Studies in Epidemiology (STROBE) guidelines (**Supplementary Checklist**).

### 2.2 Participants and thyroid testing

For patients diagnosed with any ADHD presentation, blood tests were prescribed when psychostimulant treatment was deemed necessary and both youth and parental consent were obtained. Patients in whom no pharmacological treatment was indicated did not undergo blood testing and constitute the untested comparison group described in the Results. All tested patients were medication-naïve at the time of blood sampling. Tests were performed at community laboratories of the family’s choosing and included complete blood count, C-reactive protein, creatinine, iron status, and thyroid function (free-T3, free-T4, TSH).

Inclusion criteria required complete thyroid function testing and normal TSH values according to age- and sex-specific laboratory reference intervals. Exclusion criteria were incomplete or absent thyroid testing, abnormal TSH values, and pre-existing ADHD diagnosis with ongoing pharmacological treatment at the time of assessment. Thyroid profiles were classified from free-T3 and free-T4 values relative to the reference intervals supplied by the reporting laboratory. The 2IfT3 profile was defined as free-T3 above the upper reference limit with free-T4 and TSH both within their reference intervals.

### 2.3 Clinical assessment and data collection

DSM-IV or DSM-5 criteria for ADHD, Oppositional Defiant Disorder (ODD), and Conduct Disorder (CD) were assessed during the clinical interview. Data extracted from patient files included sex, age at clinical assessment, age at blood testing, body mass index, ADHD presentation, and symptom counts for inattention, hyperactivity-impulsivity, ODD, and CD. Socioeconomic status, iodine intake, and pubertal (Tanner) staging were not systematically recorded and were therefore unavailable for analysis.

### 2.4 Laboratory methods and analytical platforms

Because samples were analyzed by community laboratories rather than a single central facility, analytical platform varied across patients. Complete assay documentation — platform, technique, and reference intervals — was available for 697 of the 731 patients with a complete thyroid panel (95.3%). The distribution was ADVIA Centaur/IECL (n = 404, 55.3%), Beckman Coulter/EIA (n = 162, 22.2%), ABBOTT Architect-Alinity/CMIA (n = 93, 12.7%), and ECLIA (n = 38, 5.2%); the remaining 34 patients (4.7%) were analyzed on platforms used too infrequently for separate adjustment, or with incomplete documentation, and were excluded from platform-adjusted models. Prior to the French Order of November 26, 1999, laboratories were not required to report immunoassay technique or reference intervals; despite efforts to retrieve missing information, documentation remained incomplete for the earliest samples. The 90% confidence intervals around reference limits were not available from any laboratory. All regression models therefore included analytical platform as a covariate.

### 2.5 Statistical analysis

Data were collected in a spreadsheet, exported to Stata^®^ version 15.1 (46), and recoded for analysis. Missing values were not imputed and no data were excluded on the basis of outlying values. All tests were two-sided with the significance threshold set at 0.05.

Between-group comparisons of categorical variables used Pearson chi-square tests. Continuous variables were compared using analysis of covariance (ANCOVA), with partial-η² reported for each term. Departures from normality were assessed with the Kolmogorov–Smirnov and Shapiro–Wilk tests and quantified by skewness and kurtosis. In the largest immunoassay subgroup, individual free-T3 values were converted to z-scores relative to published pediatric normative data (6), computed as z = (observed value − P50)/SD with SD estimated as (P97.5 − P2.5)/3.92, stratified by sex and age group.

The primary analysis was a logistic regression predicting the 2IfT3 profile from ADHD presentation, age, sex, the Sex × Age interaction, and analytical platform. The binary outcome contrasts patients with the 2IfT3 profile against those with an entirely normal thyroid profile. Forty patients (5.6%) were therefore not included in the regression models: 36 presented another abnormal profile, chiefly isolated free-T4 abnormalities or combined free-T3 and free-T4 abnormalities, and 4 lacked one or more reference limits. These patients are retained in the denominator of the prevalence estimates reported in the Results, which are therefore calculated on the full sample.

Robustness was examined by sequentially adding comorbidity diagnoses to this base model: ODD (Model 2), then ODD and CD jointly (Model 3). The continuous fT3/fT4 ratio was analyzed by linear regression with the same covariate structure, providing a measure of peripheral conversion independent of reference-interval definitions; because this measure does not require profile classification, it retains patients with other abnormal profiles, which explains its larger analytic sample. In the ancillary longitudinal analysis, logistic regression with standard errors clustered on patient identifier accounted for the non-independence of repeated measurements within individuals.

No formal correction for multiple comparisons was applied. All models tested a single pre-specified hypothesis — that the frequency of the 2IfT3 profile varies with age in a sex-dependent manner — across three complementary operationalizations of the same construct (binary classification, continuous ratio, and z-score deviation), rather than screening independent hypotheses. Applying a family-wise correction across analyses designed to converge on one question would be overly conservative. Secondary and exploratory findings should nonetheless be interpreted with this in mind, and the study is descriptive and hypothesis-generating rather than confirmatory.

Analytic samples differed across models because of missing covariate data (**Supplementary Table 6**). Of the 715 patients in the descriptive sample, 40 were excluded because their thyroid profile fell outside the binary contrast, and 29 further because their analytical platform was infrequently used or undocumented, leaving 646 children and adolescents for the base model. Documentation of comorbidity status reduced this to 556 for Model 2 and 465 for Model 3; all patients lost at these two steps had undocumented ODD or CD status respectively. Excluded patients did not differ from those retained in age (11.7 vs 11.2 years, p = 0.186) or sex distribution (χ²(1) = 1.59, p = 0.207). Together with the stability of the Sex × Age estimate across all three models, this makes differential attrition an unlikely explanation for the observed associations.

## 3 Results

### 3.1 Study sample

**Table 1** presents the patient cohort (n = 2,042; 1,468 males) who had their first consultation between March 2004 and December 2022. Among these, 993 patients had no blood test, while 1,049 underwent at least one blood test, including 121 who had a second blood test for various reasons. The sample included 171 families (defined as at least one child with at least one biological parent), 166 sibling pairs, 11 dizygotic twin pairs, and 2 monozygotic twin pairs.

**Table 1:** Age of patients at the first consultation and, when applicable, at first and second blood draw, by sex and age group, separately for patients who never underwent a blood draw and for those who underwent at least one.

Group and age variable	Younger than 6 years	6 to 17 years	18 years and older
Patients with no blood draw (N = 993)			
Age at first consultation			
Boys and men	N = 45; 5.09 ± 0.85 [1.67; 5.98]	N = 564; 10.98 ± 2.89 [6.08; 17.83]	N = 95; 32.90 ± 13.37 [18.09; 68.85]
Girls and women	N = 17; 4.90 ± 1.07 [1.73; 5.83]	N = 196; 12.51 ± 3.20 [6.12; 17.62]	N = 76; 35.66 ± 11.48 [18.03; 62.52]
Patients with at least one blood draw (N = 1,049)			
Age at first consultation			
Boys and men	N = 12; 5.34 ± 0.64 [4.26; 6.54]	N = 656; 10.88 ± 3.09 [2.16; 18.03]	N = 96; 31.72 ± 13.52 [7.86; 63.58]
Girls and women	N = 0	N = 180; 11.26 ± 3.56 [4.13; 17.86]	N = 105; 36.87 ± 11.76 [14.12; 59.56]
Age at first blood draw			
Boys and men	N = 12; 5.46 ± 0.39 [4.86; 5.89]	N = 656; 11.28 ± 3.02 [6.02; 17.98]	N = 96; 32.49 ± 12.90 [18.01; 63.61]
Girls and women	N = 0	N = 180; 11.64 ± 3.48 [6.05; 17.83]	N = 105; 37.18 ± 11.40 [18.13; 59.77]
Age at second blood draw			
Boys and men	N = 0	N = 82; 12.59 ± 2.71 [6.57; 17.70]	N = 15; 25.90 ± 9.69 [18.56; 51.39]
Girls and women	N = 0	N = 9; 14.37 ± 2.71 [9.22; 17.35]	N = 15; 28.71 ± 10.63 [18.29; 52.14]

Values are N; mean ± standard deviation [minimum; maximum].

Among 1,597 patients aged between 6 and 17 years at the first consultation, those undergoing thyroid testing did not differ in age at initial consultation from those who did not (median difference 0.30 years, 95% CI [−0.88; 0.28], p = 0.309; quantile regression). Sex was also unrelated to testing probability (χ²(1) = 3.39, p = 0.066), confirming that tested and untested groups were comparable in age and sex distributions.

**Figures 2**, **3** illustrate the distribution of patients with and without blood tests, respectively, categorized by age group at first consultation, sex, ADHD diagnosis, and presentation when applicable. Detailed thyroid profiles for patients with abnormal TSH levels are presented in **Supplementary Tables 3**, **4**.

**Figure 2:**
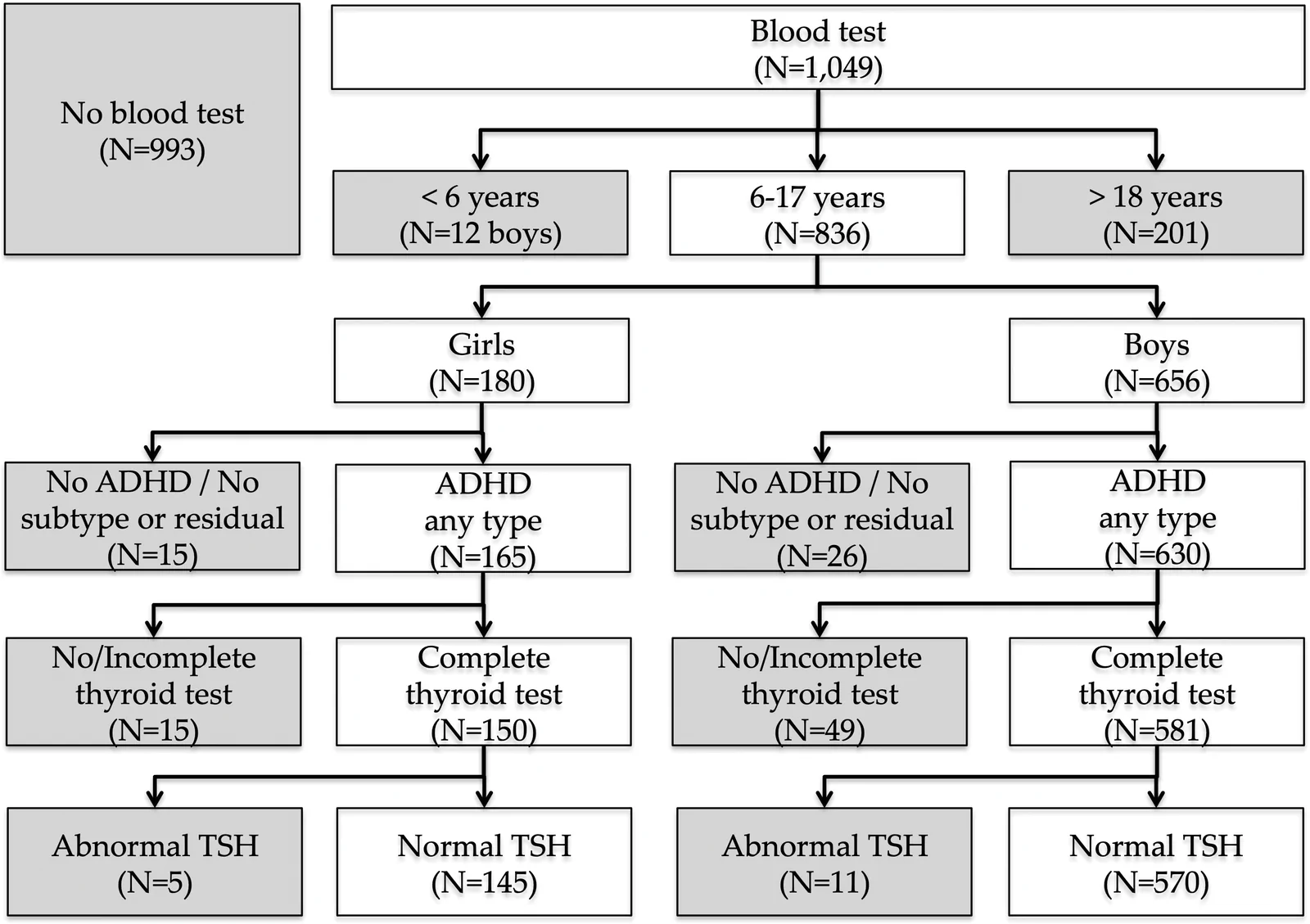
Breakdown by age group at first consultation, sex, and ADHD presentation of the 1,049 patients who underwent blood testing before methylphenidate prescription (PInattentive: Predominantly Inattentive; PHI: Predominantly Hyperactive-Impulsive; Combined).

**Figure 3:**
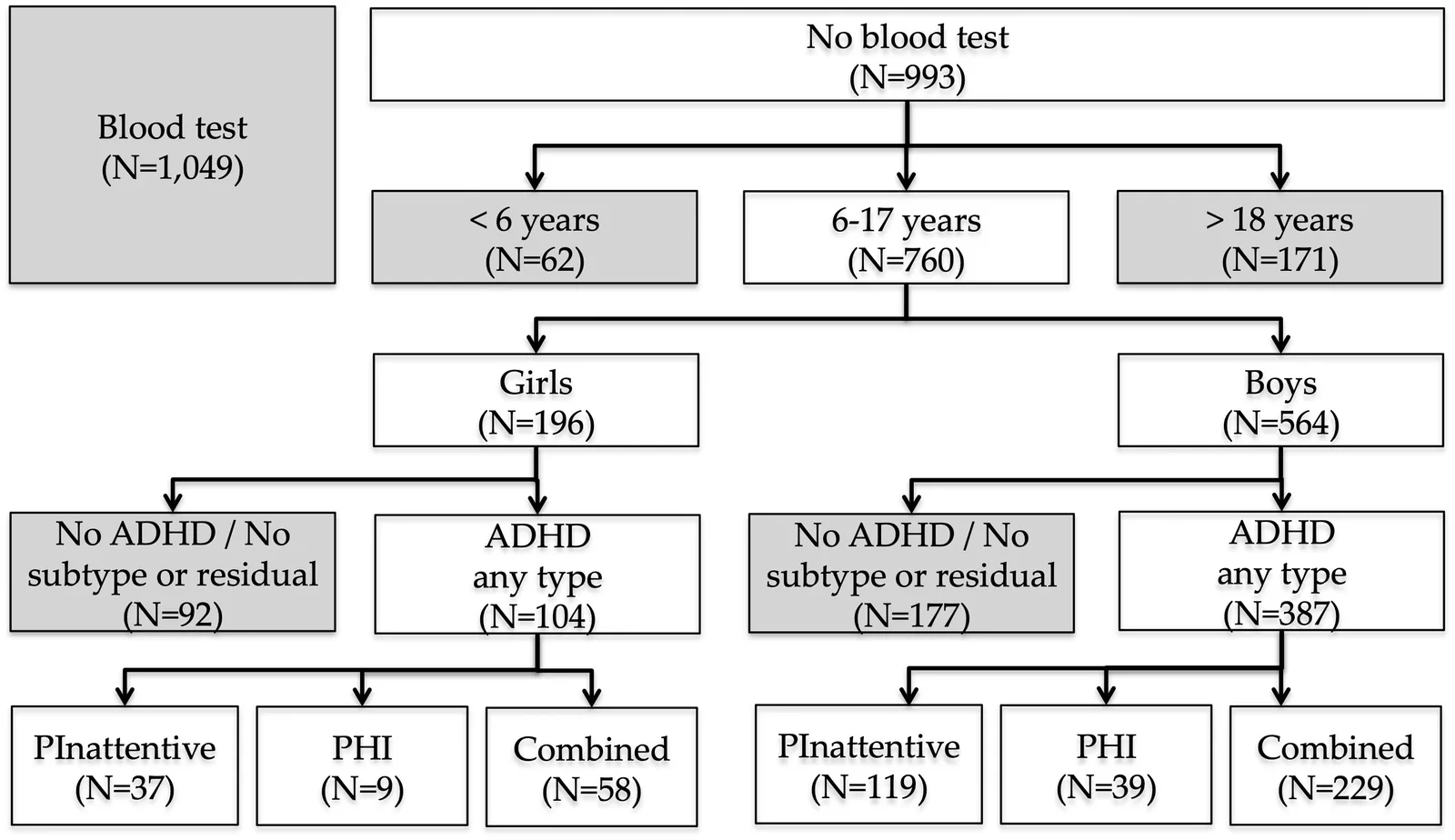
Breakdown by age group at first consultation, sex, and ADHD presentation of the 993 patients who did not undergo blood testing because methylphenidate was not prescribed (PInattentive: Predominantly Inattentive; PHI, Predominantly Hyperactive-Impulsive; Combined).

Among the 731 patients with a complete thyroid panel, the distribution by analytical platform was as follows: ADVIA Centaur/IECL (n = 404, 55.3%; 80 girls), Beckman Coulter/EIA (n = 162, 22.2%; 30 girls), ABBOTT Architect-Alinity/CMIA (n = 93, 12.7%; 24 girls), and ECLIA (n = 38, 5.2%; 8 girls).

### 3.2 Study duration

The 19-year study period was divided into three segments of equal duration. Age and sex composition varied modestly across the three segments (age: F(2,1584) = 19.44, p < 0.001, partial-η² = 0.024; sex: χ²(2) = 22.46, p < 0.001), with small effect sizes; all patients were therefore pooled for thyroid profiling analyses.

Combined presentation was overrepresented among patients undergoing blood tests (χ²(2) = 9.82, p = 0.007), particularly in the first segment (χ²(2) = 13.85, p = 0.001). Documented prevalence of both comorbidities declined over the study period — ODD from 44.8% to 36.4% (χ²(2) = 14.82, p = 0.001) and CD from 19.4% to 4.2% (χ²(2) = 30.09, p < 0.001) — while documentation completeness increased over the same period, from 34.5% to 84.0% for ODD and from 30.1% to 73.6% for CD. These trends most plausibly reflect evolving recording practices rather than a change in the clinical population. Analyses therefore used binary diagnostic status rather than symptom counts, to avoid structured missingness.

Pre-therapeutic blood test prescription showed statistical associations with inattention symptom count (OR = 1.399 [1.292; 1.514], p < 0.001), hyperactivity-impulsivity symptom count (OR = 1.066 [1.013; 1.122], p = 0.014), and consultation during the second segment (OR = 1.358 [1.004; 1.838], p = 0.047). The total ADHD symptom count yielded similar results (OR = 1.167 [1.125; 1.211], p < 0.001). However, substantial overlap in symptom profiles between tested and non-tested patients precluded identification of clear clinical thresholds for systematic testing. Combined with patient attrition before test completion, these findings indicate blood tests were prescribed on clinical judgment rather than by explicit protocol.

### 3.3 Comparison with pediatric reference intervals

Z-score analysis was performed in the ADVIA Centaur subgroup (n = 404), the most frequently used platform in this study (55.3%), for which validated pediatric reference intervals are also available (6). Z-scores were computed as z = (observed value − P50)/SD, with SD estimated as (P97.5 − P2.5)/3.92, stratified by sex and age group (6–10, 11–14, 15–18 years).

Results revealed a systematic positive shift relative to the published norms (**Supplementary Table 5**): mean z-score = +0.39 ± 0.90 SD (95% CI [+0.30; +0.48]; t(403) = 8.59, p < 0.001), with 92.2% of patients within [P2.5; P97.5] versus the expected 95%. Global distribution fit was excellent (Kolmogorov–Smirnov D = 0.002, p = 0.997), though with narrower-than-expected dispersion (SD = 0.90 vs 1.0; χ²(403) = 309.27, p = 0.004) and excess kurtosis (3.84 vs 3.0 expected), suggesting heavier tails and outlying subgroups. Sex-stratified analyses confirmed that girls showed a normal distribution (kurtosis = 3.38, Shapiro–Wilk p = 0.79) whereas boys exhibited significant excess kurtosis (3.98, p = 0.024), most pronounced in boys aged 11–14 years (n = 124, kurtosis = 4.77), who also showed the largest median elevation relative to the published norms (**Supplementary Figures I, II**).

### 3.4 The 2IfT3 thyroid profile

As shown in **Figure 2**, among 795 patients aged 6–17 years diagnosed with ADHD, 64 had incomplete or no thyroid testing and 16 had abnormal TSH levels, yielding a final sample of 715 patients (570 boys, 145 girls). Most patients were euthyroid (normal free-T4 and TSH): 95.44% of boys and 94.17% of girls. **Table 2** shows that the most common abnormality was the 2IfT3 profile (33.68% of boys, 27.59% of girls), with no sex difference (χ²(1) = 1.51, p = 0.219). All patients were medication-naïve at the time of initial thyroid assessment, ensuring that observed 2IfT3 profiles reflect untreated ADHD pathophysiology rather than medication effects.

**Table 2:** Thyroid profile of boys and girls with ADHD of any presentation and normal TSH level, classified by free-T3 and free-T4 status relative to laboratory reference intervals.

		Boys (N = 570)			Girls (N = 145)		
Free-T4	Level	Free-T3 low	Free-T3 normal	Free-T3 high	Free-T3 low	Free-T3 normal	Free-T3 high
	Low	1 (0.18%)	15 (2.63%)	5 (0.88%)	0 (0.00%)	5 (3.45%)	1 (0.69%)
	Normal	3 (0.53%)	349 (61.23%)	192 (33.68%)	4 (2.76%)	94 (64.83%)	40 (27.59%)
	High	0 (0.00%)	3 (0.53%)	2 (0.35%)	0 (0.00%)	0 (0.00%)	1 (0.69%)

Percentages are computed within sex on the full sample (570 boys, 145 girls). The 2IfT3 profile — high free-T3 with normal free-T4 — is the most frequently observed abnormality in both sexes.

The frequency of the profile among boys was stable across the first two segments of the study period (29.6% and 28.6%) and then rose sharply in the final segment (53.8%; χ²(2) = 28.65, p < 0.001), an observation we return to in the Discussion.

We conducted logistic regression (N = 646; adjusted for immunoassay platform) predicting thyroid profile from ADHD presentation, age, sex, and the Sex × Age interaction (**Table 3**). The model included 519 boys and 127 girls, of whom 187 and 40 respectively presented the 2IfT3 profile. The Sex × Age interaction was highly significant (OR = 1.36 [1.15; 1.60], p < 0.001). Among girls, each additional year reduced 2IfT3 odds by 26% (OR = 0.74 [0.63; 0.87], p < 0.001), indicating substantial developmental normalization. Among boys, no age-related change was detected (OR = 1.00 [0.93; 1.08], p = 0.936); the narrow confidence interval excludes a decline comparable to that observed in girls, but does not establish perfect stability. ADHD presentation showed no association with thyroid profile (χ²(2) = 0.19, p = 0.909).

**Table 3:** Sex-specific developmental patterns of the 2IfT3 profile: (A) clinical characteristics by sex and thyroid profile, and (B) logistic regression models predicting the 2IfT3 profile from ADHD presentation, age, sex and their interaction, with sequential addition of comorbidity diagnoses.

(A) Clinical characteristics									
	Boys					Girls			
Characteristic	All		Normal	2IfT3		All	Normal		2IfT3
**Age (years)**	N = 541; 11.15 ± 3.00		N = 349; 11.21 ± 3.09	N = 192; 11.02 ± 2.82		N = 134; 11.29 ± 3.33	N = 94; 12.12 ± 3.45		N = 40; 9.33 ± 1.97
ADHD presentation									
*Predominantly Inattentive*	155		105	50		56	42		14
*Predominantly Hyperactive-Impulsive*	29		15	14		6	5		1
*Combined*	357		229	128		72	47		25
ADHD symptoms	N = 518		N = 331	N = 187		N = 123	N = 83		N = 40
*Inattention*	7.62 ± 1.26		7.63 ± 1.29	7.60 ± 1.20		7.47 ± 1.39	7.47 ± 1.43		7.48 ± 1.30
*Hyperactivity-impulsivity*	6.32 ± 2.34		6.30 ± 2.36	6.36 ± 2.30		5.72 ± 2.49	5.57 ± 2.58		6.03 ± 2.29
*Total*	13.92 ± 2.82		13.89 ± 2.85	13.97 ± 2.77		13.19 ± 2.92	13.04 ± 2.92		13.50 ± 2.93
**ODD symptoms**	N = 418; 4.44 ± 2.05		N = 259; 4.57 ± 1.99	N = 159; 4.23 ± 2.13		N = 92; 4.12 ± 2.30	N = 63; 3.89 ± 2.22		N = 29; 4.62 ± 2.41
**CD symptoms**	N = 291; 2.08 ± 1.91		N = 174; 2.20 ± 1.89	N = 117; 1.89 ± 1.93		N = 60; 1.48 ± 1.58	N = 40; 1.30 ± 1.38		N = 20; 1.85 ± 1.90
(B) Logistic regression models									
Predictor		Model 1 (N = 646)			Model 2 (N = 556)			Model 3 (N = 465)	
Boys/girls included		519/127			450/106			376/89	
2IfT3 in boys/girls		187/40			172/33			147/28	
ADHD presentation (ref: Combined)		χ²(2) = 0.19, p = 0.909			χ²(2) = 0.86, p = 0.652			χ²(2) = 0.09, p = 0.955	
*Predominantly Inattentive*		1.24 [0.27; 5.71], p = 0.779			1.94 [0.37; 10.19], p = 0.432			1.33 [0.20; 8.70], p = 0.764	
*Predominantly Hyperactive-Impulsive*		1.65 [0.12; 22.92], p = 0.710			2.35 [0.14; 39.24], p = 0.553			0.99 [0.04; 26.05], p = 0.997	
Sex (ref: girls)		0.04 [0.01; 0.26], p < 0.001			0.09 [0.01; 0.56], p = 0.010			0.05 [0.01; 0.38], p = 0.004	
Sex × Age interaction		1.36 [1.15; 1.60], p < 0.001			1.29 [1.08; 1.53], p = 0.004			1.36 [1.12; 1.65], p = 0.002	
*Age effect in girls*		0.74 [0.63; 0.87], p < 0.001			0.79 [0.67; 0.93], p = 0.005			0.74 [0.62; 0.89], p = 0.002	
*Age effect in boys*		1.00 [0.93; 1.08], p = 0.936			1.02 [0.94; 1.11], p = 0.633			1.01 [0.93; 1.11], p = 0.789	
ODD diagnosis (vs. absent)		—			0.95 [0.65; 1.38], p = 0.781			0.94 [0.62; 1.44], p = 0.787	
CD diagnosis (vs. absent)		—			—			0.64 [0.33; 1.24], p = 0.188	
Model likelihood ratio		χ²(10) = 45.72, p < 0.001			χ²(11) = 35.05, p < 0.001			χ²(12) = 31.46, p = 0.002	
Pseudo-R²		0.055			0.048			0.051	

Values in panel A are N; mean ± standard deviation. Values in panel B are odds ratios with 95% confidence intervals. All models adjusted for immunoassay platform (IECL, EIA, CMIA, ECLIA). Age effects in boys were obtained by linear combination of the age main effect and the Sex × Age interaction. OR, odds ratio; ODD, oppositional defiant disorder; CD, conduct disorder.

To test robustness across comorbid conditions, we sequentially added comorbidity diagnoses to the base model: first ODD (Model 2, N = 556), then ODD and CD jointly (Model 3, N = 465). Neither ODD nor CD predicted thyroid profile (**Table 3**). The Sex × Age interaction remained significant across all models (OR range 1.29–1.36, all p ≤ 0.004), with girls consistently showing age-related normalization (OR range 0.74–0.79, representing a 21–26% annual reduction) and boys showing no detectable change (OR range 1.00–1.02, all p ≥ 0.633). This pattern indicates that sex-specific developmental trajectories are independent of comorbid diagnostic status (**Figure 4**).

**Figure 4:**
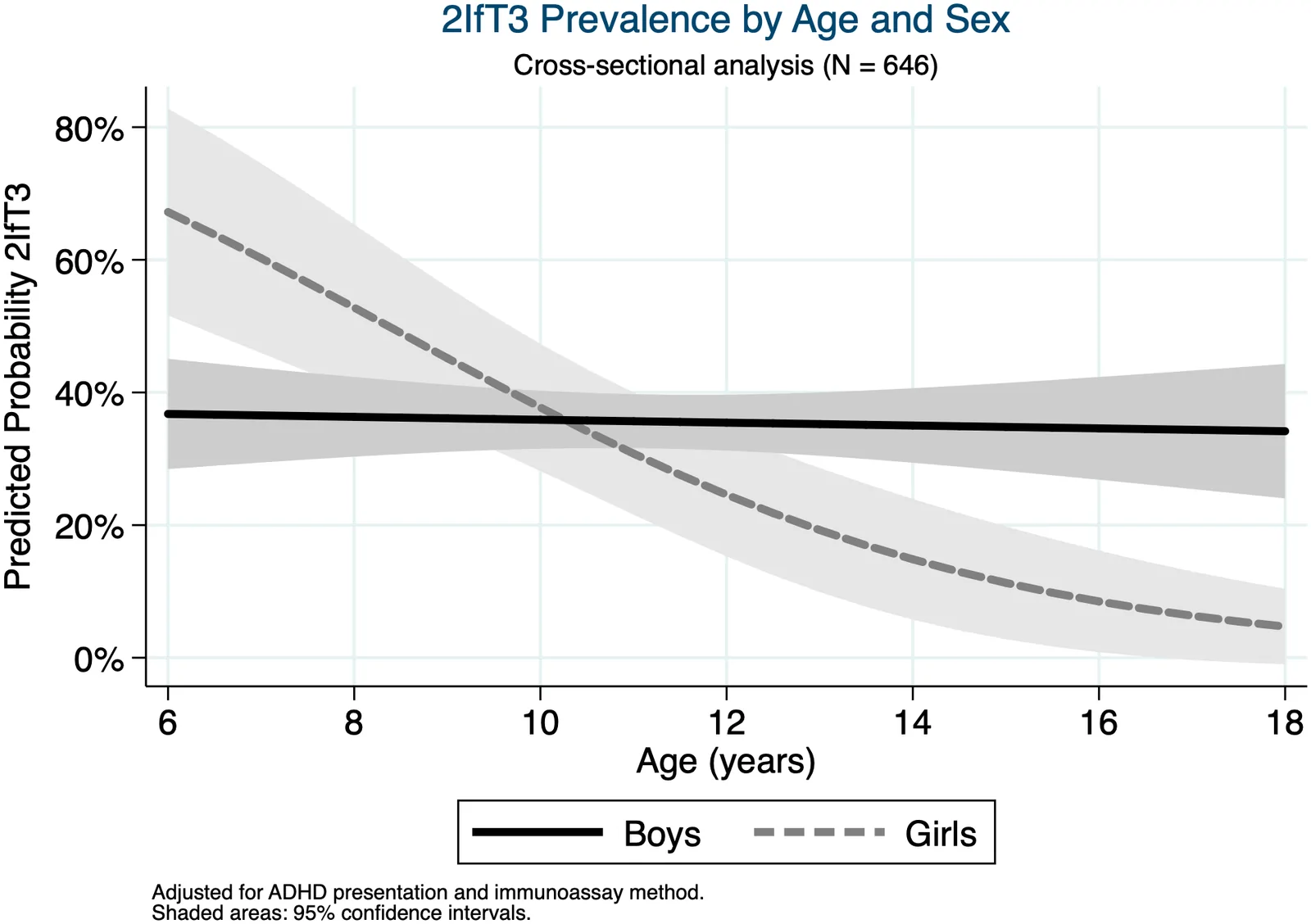
Cross-sectional 2IfT3 prevalence by age and sex (N = 646). Predicted probabilities from logistic regression adjusted for ADHD presentation and immunoassay platform. Boys: OR = 1.00 per year, p = 0.936; girls: OR = 0.74 per year, p < 0.001. Shaded areas represent 95% confidence intervals.

The analyses presented above characterized 2IfT3 prevalence across age in distinct patients, thereby capturing population-level developmental patterns. To examine whether the profile is also dynamic within individuals over time, we analyzed the subset of patients who underwent a second thyroid panel.

### 3.5 Temporal instability of the 2IfT3 profile

Among 121 patients undergoing an unplanned second blood test, 57 met inclusion criteria (age < 18 years at retest, complete thyroid assessment, normal TSH). The interval between tests ranged from 22 days to 8 years (median 2.57 years). **Figure 5** illustrates thyroid profile evolution. Among patients initially presenting 2IfT3, 53.57% (15/28) normalized while 42.86% (12/28) maintained the profile. Conversely, 50% (13/26) of initially normal patients showed 2IfT3 on retest. Three patients with initially abnormal, non-2IfT3 profiles normalized. For completeness, among 24 patients who retested when older than 18 years, 23 showed a persistent normal profile and one 2IfT3 profile appeared.

**Figure 5:**
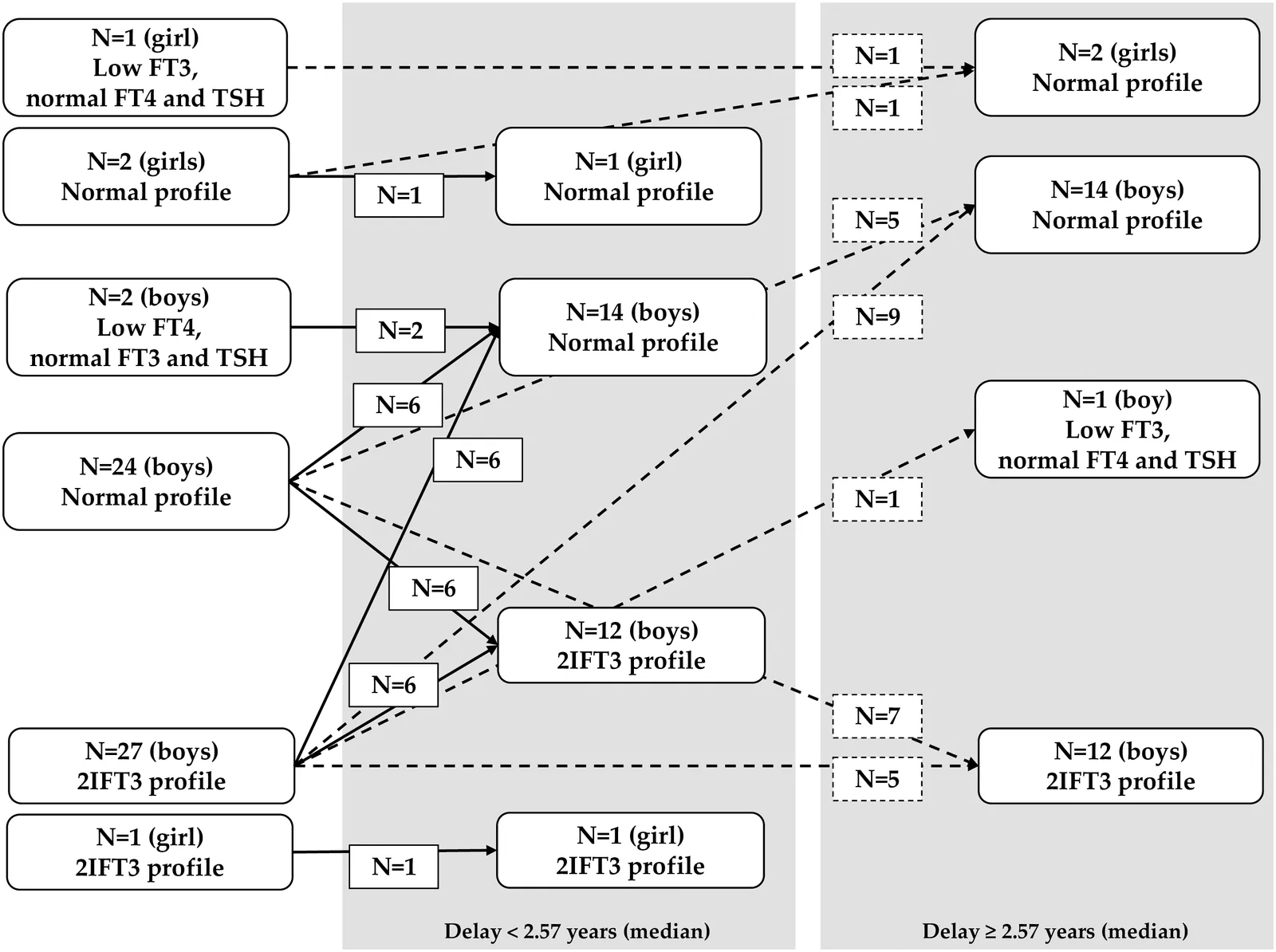
Changes in thyroid profile between baseline and retest in patients aged 6 to 17 years at both timepoints. Arrow thickness is proportional to the number of patients in each transition.

We further conducted longitudinal analyses to capture within-subject developmental change. Among 62 boys with complete repeat thyroid testing (124 observations, mean age at initial testing 10.2 years, age range 6–24 years), logistic regression with standard errors clustered on patient identifier revealed a modest age-related decrease in 2IfT3 prevalence (OR = 0.91 per year, 95% CI [0.84; 0.99], p = 0.035; **Figure 6**). While statistically significant, this effect was substantially weaker than the cross-sectional decrease observed in girls (OR = 0.74), with the confidence interval approaching unity, indicating considerable uncertainty around the magnitude of normalization in boys. Individual trajectories showed heterogeneity, with both normalizations and new appearances of the profile observed during follow-up.

**Figure 6:**
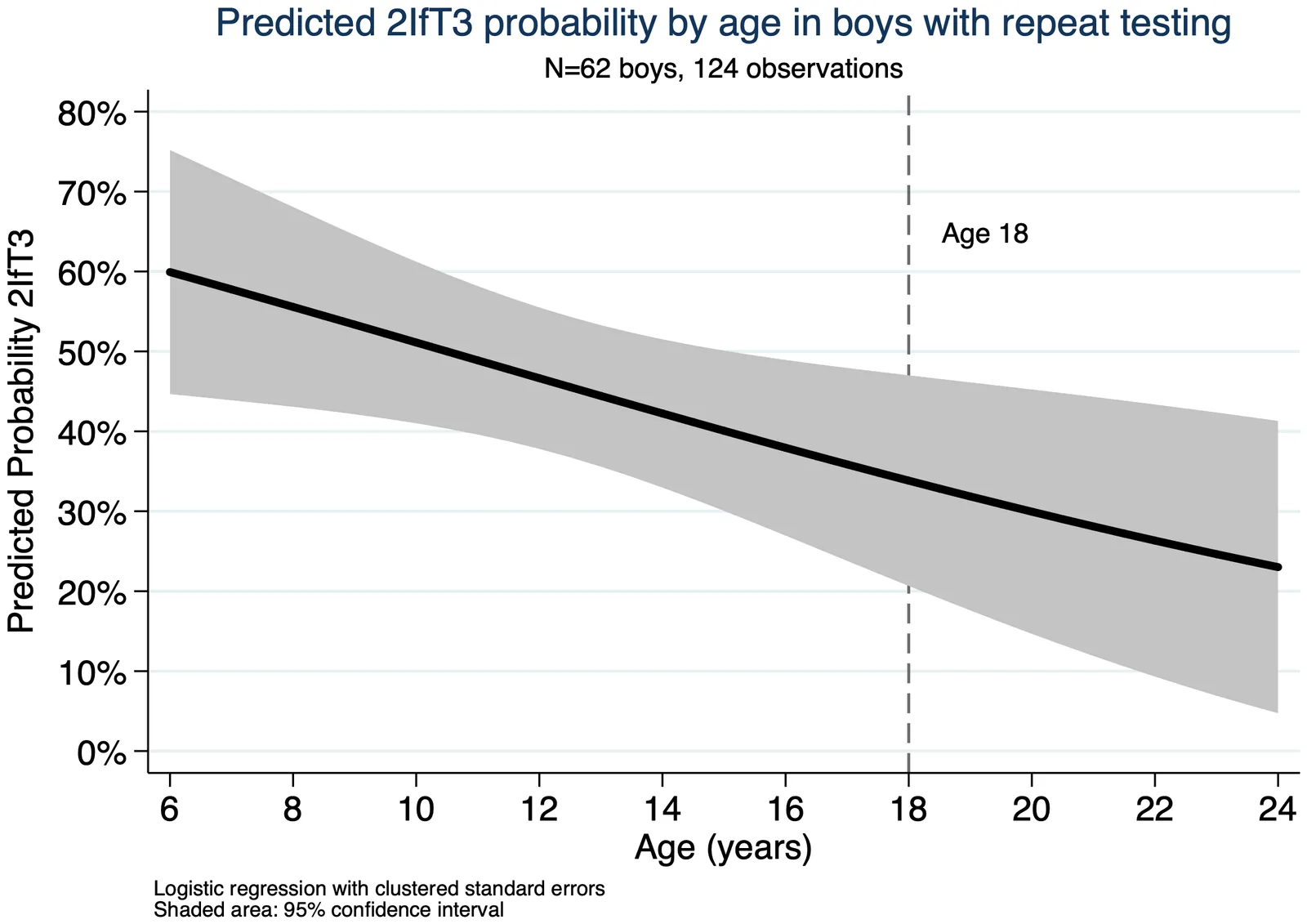
Longitudinal age-related changes in 2IfT3 probability in boys with complete repeat thyroid testing (N = 62, 124 observations). Predicted probabilities from logistic regression with standard errors clustered on patient identifier (OR = 0.91 per year, 95% CI [0.84; 0.99], p = 0.035). The shaded area represents the 95% confidence interval; the dashed vertical line marks age 18.

### 3.6 The free-T3/free-T4 ratio

To assess peripheral T4-to-T3 conversion independently of reference interval definitions, we examined the continuous fT3/fT4 ratio — a marker of deiodinase (DIO1, DIO2) activity — among 683 patients aged 6–17 years across the four main immunoassay platforms (**Table 4**). Within each platform subgroup, the ratio differed markedly between thyroid profiles, with large effect sizes (IECL: F(1,369) = 47.81, p < 0.001, partial-η² = 0.115; EIA: F(1,151) = 28.54, p < 0.001, partial-η² = 0.159; CMIA: F(1,81) = 11.29, p = 0.001, partial-η² = 0.122), confirming that the continuous measure and the binary classification capture the same underlying difference. An effect of sex was detectable only in the largest subgroup (IECL: F(1,369) = 8.36, p = 0.004, partial-η² = 0.022; EIA: p = 0.905; CMIA: p = 0.957), consistent with limited power in the smaller subgroups.

**Table 4:** Free-T3 to free-T4 ratio in patients aged 6–17 years with ADHD: (A) descriptive values and ANCOVA models within each immunoassay subgroup, and (B) three sequential linear regression models of the continuous ratio, progressively adding comorbidity diagnoses and adjusted for immunoassay platform.

(A) Descriptive values and ANCOVA models by immunoassay platform										
Platform	Profile	Sex (N)		Age (years)	fT3/fT4 ratio		Profile effect	Sex effect		Age effect
IECL	Normal	Boys (187)		11.16 ± 3.17	0.423 ± 0.057		F(1,369) = 47.81, p < 0.001, η²p = 0.115	F(1,369) = 8.36, p = 0.004, η²p = 0.022		F(1,369) = 1.06, p = 0.304, η²p = 0.003
		Girls (45)		12.44 ± 3.76	0.386 ± 0.051					
	2IfT3	Boys (118)		11.12 ± 2.69	0.457 ± 0.059					
		Girls (23)		9.43 ± 2.04	0.465 ± 0.055					
EIA	Normal	Boys (75)		11.53 ± 3.07	0.527 ± 0.088		F(1,151) = 28.54, p < 0.001, η²p = 0.159	F(1,151) = 0.01, p = 0.905, η²p < 0.001		F(1,151) = 0.10, p = 0.754, η²p = 0.001
		Girls (19)		12.48 ± 3.38	0.533 ± 0.088					
	2IfT3	Boys (51)		11.14 ± 3.28	0.618 ± 0.114					
		Girls (10)		8.67 ± 1.73	0.601 ± 0.080					
CMIA	Normal	Boys (48)		11.09 ± 3.14	0.398 ± 0.057		F(1,81) = 11.29, p = 0.001, η²p = 0.122	F(1,81) = 0.00, p = 0.957, η²p < 0.001		F(1,81) = 1.59, p = 0.211, η²p = 0.019
		Girls (15)		11.78 ± 2.87	0.374 ± 0.079					
	2IfT3	Boys (15)		10.50 ± 2.20	0.431 ± 0.051					
		Girls (7)		9.93 ± 2.09	0.479 ± 0.074					
ECLIA	Normal	Boys (22)		10.13 ± 2.41	0.387 ± 0.059		— ^a^	— ^a^		—^a^
		Girls (8)		10.68 ± 2.98	0.388 ± 0.076					
	2IfT3	Boys (3)		9.76 ± 3.41	0.426 ± 0.110					
		Girls (0)		—	—					
(B) Linear regression models of the fT3/fT4 ratio										
Predictor			Model 1 (N = 683)			Model 2 (N = 587)			Model 3 (N = 488)	
ADHD presentation (ref: Combined)			F(2,674) = 1.13, p = 0.325			F(2,577) = 0.55, p = 0.577			F(2,477) = 0.43, p = 0.653	
Sex (ref: girls)			−0.0533 [−0.1077; 0.0012], p = 0.055			−0.0503 [−0.1112; 0.0106], p = 0.105			−0.0572 [−0.1240; 0.0095], p = 0.093	
Sex × Age interaction			0.0063 [0.0016; 0.0109], p = 0.008			0.0061 [0.0009; 0.0113], p = 0.022			0.0067 [0.0012; 0.0122], p = 0.018	
*Age effect in girls*			−0.0041 [−0.0081; −0.0001], p = 0.047			−0.0035 [−0.0080; 0.0011], p = 0.132			−0.0036 [−0.0084; 0.0012], p = 0.138	
*Age effect in boys*			0.0022 [−0.0001; 0.0045], p = 0.066			0.0026 [0.0000; 0.0052], p = 0.049			0.0030 [0.0003; 0.0058], p = 0.031	
ODD diagnosis (vs. absent)			—			0.0038 [−0.0102; 0.0177], p = 0.597			0.0084 [−0.0068; 0.0236], p = 0.279	
CD diagnosis (vs. absent)			—			—			−0.0217 [−0.0448; 0.0013], p = 0.065	
Immunoassay: EIA (vs. IECL)			0.1291 [0.1144; 0.1438], p < 0.001			0.1299 [0.1137; 0.1461], p < 0.001			0.1177 [0.1005; 0.1348], p < 0.001	
Immunoassay: CMIA (vs. IECL)			−0.0190 [−0.0371; −0.0009], p = 0.039			−0.0188 [−0.0386; 0.0010], p = 0.062			−0.0247 [−0.0454; −0.0040], p = 0.020	
Immunoassay: ECLIA (vs. IECL)			−0.0392 [−0.0669; −0.0115], p = 0.006			−0.0408 [−0.0706; −0.0109], p = 0.008			−0.0578 [−0.0920; −0.0236], p = 0.001	
Adjusted R²			0.354			0.353			0.346	

Values are mean ± standard deviation in panel A, and unstandardized regression coefficients with 95% confidence intervals in panel B.

^a^ The ECLIA subgroup contained no girls with the 2IfT3 profile; the ANCOVA design is therefore incomplete and no inferential statistics are reported for this subgroup. IECL, immunoelectrochemiluminescence assay; EIA, enzyme immunoassay; CMIA, chemiluminescent microparticle immunoassay; ECLIA, electrochemiluminescence immunoassay; ODD, oppositional defiant disorder; CD, conduct disorder; η²p, partial eta squared.

Three sequential linear regression models, progressively adding ODD then CD diagnoses, consistently revealed a significant Sex × Age interaction (β range 0.0061–0.0067, all p ≤ 0.022), independent of ADHD presentation and comorbidities. The sex-specific slopes were directionally consistent across all three models — a decrease in girls and an increase in boys — although individual slopes reached significance only for girls in the base model (β = −0.0041 per year, p = 0.047) and for boys in the two comorbidity-adjusted models (β = +0.0026, p = 0.049 and β = +0.0030, p = 0.031). The convergence across analytic approaches therefore concerns the interaction itself rather than either slope in isolation. CD showed a negative association with the ratio that did not reach significance (β = −0.0217 [−0.0448; 0.0013], p = 0.065); ODD was non-significant across all models.

Critically, immunoassay platform exerted effects an order of magnitude larger than any physiological variable: EIA produced values 0.118–0.130 units above the IECL reference (p < 0.001), while CMIA and ECLIA yielded significantly lower values — demonstrating that analytical heterogeneity persists even when reference interval dependency is eliminated through ratio analysis. **Table 5** summarises the convergence of the Sex × Age effect across the three analytic approaches used in this study.

**Table 5:** Convergence of the sex × age effect across the three analytic approaches used in this study.

Analytic feature	Binary 2IfT3 classification	Continuous fT3/fT4 ratio	Free-T3 z-scores
Analytic sample	N = 646	N = 683	N = 404 (ADVIA Centaur)
Model	Logistic regression	Linear regression	Comparison with published norms
Effect metric	Odds ratio	Regression β	Mean z-score
Sex × Age interaction	1.36 [1.15; 1.60], p < 0.001	0.0063 [0.0016; 0.0109], p = 0.008	Not modelled; stratified by sex and age
Age effect, girls	0.74 [0.63; 0.87], p < 0.001	−0.0041 [−0.0081; −0.0001], p = 0.047	Normal distribution (kurtosis 3.38)
Age effect, boys	1.00 [0.93; 1.08], p = 0.936	0.0022 [−0.0001; 0.0045], p = 0.066	Excess kurtosis (3.98, p = 0.024); peak at 11–14 y
Robustness to comorbidity	Interaction significant in all 3 models	Interaction significant in all 3 models	Not applicable
Reference-interval dependent	Yes	No	Yes (external norms)
Residual platform effect	Adjusted	Persists: EIA +0.129 vs IECL, p < 0.001	Eliminated by design

All models adjusted for immunoassay platform except the z-score analysis, restricted by design to a single platform. Analytic samples differ because the continuous ratio does not require profile classification and therefore retains patients with other abnormal profiles. OR, odds ratio; β, unstandardized regression coefficient.

## 4 Discussion

The 2IfT3 profile is physiologically paradoxical: increased free-T3 would ordinarily suppress TSH through negative feedback, yet 33.68% of boys and 27.59% of girls displayed free-T3 above the upper reference limit while both TSH and free-T4 levels remained within their reference intervals — an order of magnitude above the 2.5% expected by construction of a reference interval. Three considerations must frame everything that follows. First, no non-ADHD comparison group was available, so this study describes the frequency of a biochemical profile within an ADHD cohort and cannot establish that the profile is specific to ADHD. Second, the profile proved markedly unstable on retest, which constrains any biomarker interpretation. Third, the retrospective design carries several sources of bias and biological uncertainty that must be weighed before any mechanistic reading. We address these in turn before considering possible mechanisms, which we present as hypothesis-generating rather than established.

### 4.1 Absence of a comparison group

The absence of a non-ADHD control group is the most fundamental limitation of this study. It precludes determining whether the 2IfT3 profile and its sex-specific developmental trajectories are ADHD-related or simply reflect general population patterns.

This question is not trivial, because population-based studies in healthy children document sexually dimorphic free-T3 trajectories that resemble our findings: girls show progressive free-T3 decrease throughout childhood and adolescence, while boys exhibit a transient mid-adolescent peak before decreasing (47, 48). The normalization we observed in girls is directionally consistent with these healthy population patterns. The stability we observed in boys across ages 6–24 contrasts with the peak-then-decrease described in healthy adolescent males (47), but this contrast cannot be interpreted without matched controls. The absence of pubertal staging compounds the uncertainty: puberty is accompanied by a temporary free-T3 peak in males (8), and whether the boys aged 11–14 years who drove the largest z-score deviations are experiencing an exaggerated version of this normative peak, or a distinct trajectory, cannot be determined from our data.

Three questions therefore remain open, and none can be resolved here: whether the approximately 30% frequency of 2IfT3 exceeds baseline population rates; whether the normalization observed in girls reflects ADHD-related maturation or tracks universal female developmental patterns; and whether the pattern observed in boys reflects ADHD pathophysiology or differences in developmental timing. Case-control studies applying identical assays and age- and sex-specific reference intervals to both groups are required.

### 4.2 Sources of bias

Blood tests were ordered only for patients aged 6 years or older with confirmed ADHD in whom stimulant treatment was considered, creating selection toward more severe presentations; tested and untested patients did not, however, differ in age or sex ratio. All assessments were conducted by a single clinician over two decades using DSM-IV or DSM-5 criteria, blind to thyroid results. Temporal shifts in documented diagnostic patterns — Combined presentation more frequent early, comorbidity documentation becoming more complete while documented prevalence declined — reflect evolving assessment and recording conventions rather than changes in the clinical population; analyses used binary diagnostic status rather than symptom counts to avoid structured missingness. Socioeconomic status, iodine intake, and BMI were not systematically collected.

### 4.3 Uncertainties in the biological data

TBG (thyroxine-binding globulin) levels and iodine status were not assessed. France is classified as iodine-sufficient on national median urinary iodine concentrations (49), recruitment spanned two decades without clustering of clinical thyroid signs, and TSH was normal in 96.67% (754/780) of the overall sample, which argues against systematic iodine-related confounding. Transient T3 toxicosis can accompany Graves’ disease, which none of our patients had.

The lack of control over immunoassay platforms is a major limitation, as patients used their preferred community laboratories, with the ADVIA Centaur accounting for more than half of measurements. Inter-assay discrepancies in free-T3 and free-T4 levels are well documented (50), with systematic deviations from the LC-MS/MS reference standard (51, 52). These differences have not been acknowledged in the ADHD-thyroid literature, despite their potential to confound prevalence estimates and cross-study comparisons. Laboratories also varied in technique documentation, applied stratified RIs without specifying stratification criteria, and reported values with differing precision and units, even when using identical platforms. Our statistical adjustment for platform is a partial remedy and underscores the need for assay standardization in this field.

Reference intervals (RIs) compound this. In pediatrics, RIs vary throughout development, yet manufacturers often apply adult-derived intervals to children without explicit validation. Whether the 2IfT3 profile represents a true physiological deviation or misclassification arising from inappropriate reference standards cannot be resolved with current data. To reduce this dependency we examined the continuous fT3/fT4 ratio, a platform-adjusted measure of peripheral conversion independent of RI definitions; it reproduced the same sex-specific interaction observed with the binary classification. Relatedly, the frequency of the 2IfT3 classification among boys rose from 29.6% and 28.6% in the first two segments of the study period to 53.8% in the final segment (χ²(2) = 28.65, p < 0.001), despite minimal change in the continuous ratio. This suggests that more boys crossed classification thresholds without substantial quantitative hormonal shifts — plausibly reflecting progressive adoption of pediatric and sex-specific reference intervals by community laboratories rather than a biological trend.

The largest primary cohort published to date reported reduced free-T4, TT3, and TT4 with unremarkable free-T3 in ADHD, a pattern superficially at odds with ours (53). ADHD patients in that cohort were, however, significantly older than controls (7.24 ± 2.24 vs 6.34 ± 3.62 years, p < 0.001) and more frequently male (81.4% vs 57.7%, p < 0.001), and between-group comparisons were not adjusted for these two well-established determinants of pediatric thyroid hormone levels. The discrepancy may therefore be methodological rather than biological. In the present study, the availability of validated pediatric normative data for the ADVIA Centaur platform (6) allowed individual free-T3 values to be expressed as age- and sex-specific z-scores in the largest immunoassay subgroup (n = 404, 55.3%).

### 4.4 Reconciling the cross-sectional and longitudinal findings

Cross-sectional absence of an age effect in boys (OR = 1.00) and a modest longitudinal decrease (OR = 0.91, 95% CI [0.84; 0.99], p = 0.035) are not contradictory. The weak longitudinal effect emerges from bidirectional movement: some boys normalized over time while others acquired the profile during follow-up, normalizations only slightly outnumbering new appearances. This simultaneously explains the modest net decrease and its compatibility with cross-sectional stability, and contrasts with the unidirectional normalization observed in girls (OR = 0.74).

Reconciling a frequency of approximately 30% with switching in about half of retested patients is the deeper interpretive challenge. Three non-exclusive explanations are plausible. Analytical variability near reference boundaries could produce apparent switches without biological change; this is credible for borderline values but does not account for the sex-specific trajectories. The profile may be state-dependent, reflecting transient metabolic responses to stress, sleep disruption, symptom exacerbation, or developmental transitions; notably, a prospective 12-month study found no thyroid changes associated with low-dose methylphenidate or atomoxetine in 118 drug-naive children (54), making pharmacotherapy an unlikely explanation. Finally, the cohort may be heterogeneous, some patients carrying stable trait markers and others showing fluctuating state-dependent responses. Retests here were unplanned, occurred at variable intervals, and lacked coordinated symptom assessment, so none of these can be tested with our data.

### 4.5 Mechanistic hypotheses

The following hypotheses are testable rather than established, and the analytical variability described above cautions against overreading any single result.

The sex-specific trajectories we observed, in both the binary classification and the continuous ratio, track patterns documented in healthy pediatric populations, where girls show progressive free-T3 decrease from childhood through mid-adolescence and boys display a transient peak around age 14 (47, 48). Whether ADHD youths experience an exaggerated version of a normative trajectory, or something distinct, cannot be answered without controls. The continuous fT3/fT4 ratio, reflecting integrated deiodinase activity, confirmed a sexually dimorphic pattern through a robust Sex × Age interaction (β = 0.0061–0.0067, all p ≤ 0.022), independent of ADHD presentation and comorbidities. However, inter-immunoassay heterogeneity — EIA yielding values 0.118–0.130 units above IECL, an order of magnitude larger than any physiological effect — critically undermines quantitative interpretation, and ratio-based approaches did not eliminate it, indicating non-proportional systematic biases affecting free-T3 and free-T4 differentially.

A hormonal basis for these patterns is plausible: growth hormone enhances DIO2 with sexually dimorphic patterns (55), testosterone increases hepatic DIO1 in animal models (56), and estradiol increases DIO3 *in vitro (*57). Whether enhanced T3 conversion in ADHD represents a compensatory mechanism supporting neuronal energy demands or contributes to pathophysiology remains unclear; the temporal instability we observed argues against fixed enzymatic dysfunction and points toward dynamic modulation of deiodinase activity. Future studies should employ LC-MS/MS to eliminate the inter-method noise that currently makes mechanistic conclusions premature.

Transporter variants offer an alternative but less compatible account. MCT8, encoded by SLC16A2, is the principal T3 transporter (58); severe mutations cause Allan-Herndon-Dudley syndrome, characterized by intellectual disability and motor dysfunction (59, 60), features absent in our sample. Milder functional variants could alter tissue-specific T3 availability without the full phenotype, but would presumably generate stable rather than fluctuating profiles. The bidirectional switching we documented therefore argues against fixed genetic determinants as the primary mechanism, though variants could modulate susceptibility to environmentally driven switches.

Metabolically, increased free-T3 raises peripheral metabolism and oxygen consumption, potentially enhancing reactive oxygen species production (61). Oxidative stress has been implicated in ADHD (62, 63), and a meta-analysis reported slightly increased oxidative stress with larger effects in younger children (64), although those studies did not assess thyroid function. If 2IfT3 profiles fluctuate over time, oxidative stress markers should fluctuate in parallel; coordinated longitudinal measurement would test this directly.

### 4.6 A hypothesis: convergence with stress-related and externalizing literatures

This section is analogical and hypothesis-generating. No criminal-justice, forensic, psychopathy, or trauma-exposure data were collected in the present cohort, and no association between the 2IfT3 profile and any such outcome was tested or found here. The literatures discussed below concern different populations, mostly adults, and are invoked only to motivate future work.

Increased T3 has been reported in two bodies of literature rarely connected to ADHD. Longitudinal Swedish studies spanning three decades found increased total-T3 with normal or decreased T4 in juvenile delinquents, persisting into adulthood specifically among those who continued offending (19, 20), and most marked in subjects with multiple juvenile offenses and childhood attention difficulties (25). Separately, euthyroid combat veterans with PTSD displayed increased T3 decades after exposure (12), a finding subsequently reported in other veteran samples and in women with non-combat trauma (10, 11, 13–17, 65) (**Supplementary Table 1**); methodological quality is heterogeneous across these reports, and the two Croatian studies share a research network and assay (10, 11) and cannot be treated as independent replications.

The pattern described in those literatures and the 2IfT3 profile share selective T3 increase with normal TSH and free-T4 and no overt thyrotoxicosis. They differ on two points: our pediatric profile is restricted to free-T3 rather than total-T3, suggesting a more localized perturbation of peripheral deiodination, and it shows a strong sex restriction with no direct counterpart in the PTSD literature. Because childhood ADHD was rarely assessed in the earlier cohorts, and because those studies did not examine individual values against age- and sex-specific reference intervals, it is possible that unrecognized ADHD was an unmeasured variable in that work. This is a hypothesis requiring systematic co-assessment of ADHD, trauma exposure, and thyroid function in appropriately designed prospective studies; nothing in the present data tests it.

## 5 Limitations

The principal limitations of this study, detailed in the preceding sections, are summarized here for clarity and completeness.

The absence of a non-ADHD control group is the most fundamental limitation. It precludes determining whether the 2IfT3 profile and its sex-specific trajectories represent ADHD-related patterns or general population dynamics. Comparison with published pediatric reference data partially mitigates but does not resolve this, since those norms derive from a different population, measured on a single platform, at a different period.

The retrospective single-center design, with thyroid testing prescribed on clinical judgment rather than by systematic protocol, introduces selection toward more severely symptomatic patients and limits generalizability. The marked sex imbalance of the cohort (570 boys, 145 girls) means that estimates for girls rest on a substantially smaller sample and should be interpreted with corresponding caution.

Pubertal staging was not available. Chronological age is only an indirect proxy for pubertal status, and interindividual variability in pubertal timing is considerable at the ages where we observed the largest deviations. Whether the boys aged 11–14 years who drove those deviations were experiencing an exaggerated form of the normative peripubertal free-T3 peak, or a distinct trajectory, cannot be established from these data. All statements about puberty in this manuscript should be read as referring to the age range broadly corresponding to pubertal development, not to directly assessed pubertal stage.

Comorbidity status was documented in only 65% of eligible patients for ODD and 52% for CD, with completeness increasing over the study period. Models 2 and 3 should therefore be read as sensitivity analyses on a selected subsample rather than as estimates generalizable to the full cohort.

Although the cohort included 171 families, 166 sibling pairs, and 13 twin pairs, thyroid testing was performed only in the index patients prior to methylphenidate initiation; parents and unaffected siblings were not tested, precluding family-based analyses of thyroid parameters, and the number of twin pairs was in any case far below that required for heritability estimation.

Body mass index, iodine status, sample collection time, and fasting status were not systematically recorded; biotin interference, although uncommon at therapeutic doses, cannot be formally excluded for some assays. Free-T3 immunoassays show well-documented inter-platform heterogeneity and systematic deviations from the LC-MS/MS reference standard, with effects an order of magnitude larger than any physiological variable observed here — an analytical limitation we addressed by adjustment but did not eliminate.

Longitudinal data were sparse and unplanned, with heterogeneous retesting intervals and indications, precluding analysis of whether profile changes tracked clinical course. Finally, comparisons with the forensic and PTSD literatures are complicated by their predominant use of total rather than free T3, and by the fact that concurrent TSH and free-T4 values are rarely reported in sufficient detail to identify isolated free-T3 increases.

These limitations motivate the registered prospective protocol ThyrADHD (ClinicalTrials.gov NCT05080491), designed to address each of them in 400 youths with ADHD through standardized UPLC-MS/MS measurement of free-T3, free-T4 and reverse-T3 alongside four immunoassay platforms, with Tanner staging, body mass index, iodine status, fasting morning sampling, appropriate comparison groups, and coordinated symptom assessment.

## 6 Conclusion

In this study of 715 medication-naïve children and adolescents with ADHD, approximately one-third presented with isolated increased free-T3 while remaining euthyroid by conventional criteria. The profile showed pronounced sex-specific patterns: girls demonstrated robust age-related normalization (OR = 0.74 per year) while boys showed no detectable age-related change (OR = 1.00), these trajectories being independent of ADHD presentation and comorbidities and reproduced by a continuous, reference-interval-independent measure of peripheral conversion. Marked temporal instability on retest — approximately half of retested patients switching profiles bidirectionally — currently precludes use as a stable biomarker. Routine free-T3 screening in ADHD therefore remains a research question rather than a clinical recommendation.

Because no concurrent non-ADHD comparison group was available, these findings describe the frequency and developmental variation of a biochemical profile within an ADHD cohort; they do not establish that the profile is specific to ADHD. Two hypotheses follow and require testing rather than assertion. First, increased T3 has been reported in stress-related and externalizing populations in which childhood ADHD was rarely assessed, raising the possibility that the profile indexes a shared neuroendocrine pattern rather than ADHD-specific pathophysiology; no data of that kind were collected here. Second, the profile’s dissociation from ADHD symptom counts, together with its frequency in medication-naïve youth, suggests it may index a metabolic state worth examining in treatment-response studies.

Whether the 2IfT3 profile is specific to ADHD or transdiagnostic remains open. A recent LC-MS/MS study in 788 autistic children found no difference in the T4:T3 or FT4:FT3 ratio compared with controls (66), contrasting with the isolated free-T3 increase observed here. If confirmed, such divergence would argue against pooling ADHD, autism, and tic disorders into a single neurodevelopmental category for thyroid analyses, and in favor of disorder-specific characterization. Resolving these questions requires prospective designs with standardized measurement and appropriate controls, which the ThyrADHD protocol is intended to provide.

## Data Availability

The datasets analyzed during the current study are not publicly available because they contain data extracted from medical records. Under the CNIL MR-004 reference methodology governing this study, access is restricted to persons involved in the research. Requests may be addressed to the corresponding author and are subject to approval by the data controller and to compliance with applicable French data protection regulations.
