# Supplementary material for: Isolated increased free-triiodothyronine in medication-naïve children and adolescents with ADHD: a retrospective observational study
Source: Front Endocrinol (Lausanne). 2026 Oct 9;17:1915311. doi: 10.3389/fendo.2026.1915311 (PMC13690655; doi:10.3389/fendo.2026.1915311)
Supplement: Supplementary file 1 [file DataSheet1.pdf]

Supplementary Table 1 — Thyroid profiles in patients with PTSD (Post-Traumatic Stress Disorder). Conversion to International System units was performed with https://unitslab.com/. Values are mean (SD). Legend: CAPS (Clinician-Administered PTSD Scale), CES (Combat Exposure Scale), LIA (Luminoimmunochemical assay), NR (Not Reported), RIA (Radioimmunoassay), SEM (Standard Error of the Mean).

Year	First author	Diagnostic	Sample (% men)	Age	Technique	TT3 (nmol/L)	Free-T3 (pmol/L)	TT4 (nmol/L)	Free-T4 (pmol/L)	TBG (nmol/L)	TSH (mIU/L)	TT3/fT4
**1994**	Mason J	DSM-III-R<br>Mississippi Scale<br>CAPS	4 x 24 PTSD<br>(100)	42.5<br>(N=96)	RIA	2.7<br>(0.59)	5.11<br>(1.08)	110<br>(23.5)	20.0<br>(3.92)	376<br>(127.4)	1.84<br>(0.78)	—
		CES	24 Controls	38 (N=24)		2.0<br>(0.39)	4.06<br>(0.73)	87<br>(17.6)	20.0<br>(4.90)	252<br>(63.7)	1.75<br>(0.69)	—
**1996**	Mason J	DSM-III-R<br>Revised PTSD Scale<br>(Solomon 1993)	11 PTSD<br>(100)	27-54	RIA	2.47<br>(0.42)	4.81<br>(0.86)	121.90<br>(16.22)	18.79<br>(2.32)	522.22<br>(94.44)	1.83<br>(1.35)	112.3<br>(28.4)
		Impact of Events Scale<br>(Horowitz 1979)	11 Controls	Matched		2.08<br>(0.24)	4.69<br>(0.78)	111.21<br>(18.92)	17.12<br>(3.09)	492.5<br>(75.93)	2.28<br>(1.60)	105.8<br>(22.1)
**1999**	Wang S	Mississippi Scale<br>score>107	12 PTSD<br>(100)	71.4	RIA	2.72<br>(0.45)	5.30<br>(0.59)	106.84<br>(15.18)	16.86<br>(2.67)	587.04<br>(121.25)	2.21<br>(1.42)	138<br>(32.6)
			18 Controls	72		2.34<br>(0.26)	4.62<br>(0.52)	108.13<br>(20.75)	17.38<br>(2.73)	531.48<br>(136.71)	1.46<br>(1.23)	115<br>(22.5)
**2002**	Kozaric-Kovacic D	DSM-IV<br>Watson's PTSD Questionnaire<br>Mississippi Scale	38 PTSD<br>(100)	35.9<br>(7.5)<br>[23; 53]	LIA	2.04<br>(0.32)	5.92<br>(1.11)	89.0<br>(20.11)	15.57<br>(2.77)	—	1.19<br>(0.76)	—
			32 Controls	36.5<br>(8.3)<br>[25; 50]		1.55<br>(0.33)	5.46<br>(0.33)	91.92<br>(26.2)	15.07<br>(2.83)	—	1.09<br>(0.63)	—
**2004**	Karlovic D	DSM-IV<br>Watson's PTSD Questionnaire<br>CAGE Questionnaire	43 PTSD<br>(100)	38.7<br>(6.2)<br>[24; 49]	LIA	2.1<br>(0.4)	5.8<br>(0.9)	88.2<br>(18.3)	15.7<br>(2.6)	—	1.3<br>(0.6)	—
			41 PTSD+AD<br>(100)	40.7<br>(5.9)<br>[26; 47]		2.1<br>(0.3)	5.8<br>(1.1)	89.3<br>(16.9)	16.4<br>(6.5)	—	1.3<br>(0.7)	—
			39 Controls	39.2<br>(6.6)		1.7<br>(0.4)	5.5<br>(0.6)	90.5<br>(25.9)	16.0<br>(2.8)	—	1.2<br>(0.5)	—

				[29; 45]								
**2005**	Friedman MJ	DSM-IV (SCID)	63 PTSD-CSA	39.8	RIA	2.44	4.18	106.07	16.48	603.70	1.45	126.93
			(0)	(9.3)		(0.41)	(0.81)	(20.47)	(2.70)	(158.15)	(0.72)	(28.60)
		CAPS										
			42 Controls	42.4		2.13	3.96	106.07	16.73	589.07	1.76	110.16
				(12.2)		(0.35)	(0.65)	(19.18)	(2.96)	(122.22)	(0.90)	(29.39)

Note. Dispersion format. Standard deviations for Mason et al. (1994) and Wang & Mason (1999) were reconstructed from the published SEM as SD = SEM × √n. Wang & Mason (1999) explicitly report mean (SEM); Mason et al. (1994) does not specify the dispersion format, but the SEM interpretation is inferred from (i) the implausibly low coefficients of variation (2–4 %) that a SD interpretation would imply for thyroid hormones at n = 96, and (ii) consistency with the same research group's conventions in subsequent publications (Wang et al. 1997; Wang & Mason 1999). Mason et al. (1996) explicitly report mean (SD); these values are tabulated as published. **Suspected typographical errors.** Two implausibly extreme standard deviations are noted: FT3 in controls of Kozaric-Kovacic et al. (2002), reported as 0.33 pmol/L (CV = 6.0 %, n = 32), and FT4 in the PTSD+AD group of Karlovic et al. (2004), reported as 6.5 pmol/L (CV = 39.6 %, n = 41) — substantially higher than for the PTSD-only group (CV = 16.6 %) and controls (CV = 17.5 %) in the same study. Typographical errors in the original publications are suspected but could not be confirmed; values are tabulated as published. **Medication status.** Mason et al. (1994) imposed a 3-week medication washout before sampling. Kozaric-Kovacic et al. (2002) and Karlovic et al. (2004), originating from the same research network in Zagreb and using the same luminoimmunochemical assay kits (Johnson & Johnson Clinical Diagnostic Products, Amersham, UK), enrolled patients all receiving psychopharmacotherapy at the time of sampling — predominantly SSRIs and benzodiazepines — without any documented washout. Friedman et al. (2005) reported antidepressant use in 36.5 % of PTSD-CSA (i.e., Childhood Sexual Abuse) participants and statistically controlled for it through ANCOVA, with the TT3 elevation persisting after adjustment. **Methodological note on Friedman et al. (2005).** This is the only female-only and non-combat-related PTSD study in the table; trauma history was childhood sexual abuse. Thyroid assays were performed in the laboratories of J. Mason and S. Wang (West Haven VA, CT) using the same RIA kits as Mason et al. (1994) and Wang & Mason (1999), ensuring inter-study analytical consistency. The authors documented an unusual reduction in TSH (rather than the elevation or no change seen in male combat samples) and absence of TBG elevation, both persisting after adjustment for antidepressant use, lifetime PTSD, prior trauma history, and depressive symptoms.

Supplementary Table 2 — Review of studies reporting thyroid function in children and adolescents with ADHD. Conversion to International System units was performed via https://unitslab.com. **Diagnostic and rating instruments:** CATRS, Conners' Abbreviated Teacher Rating Scale; DISYPS-II, Diagnostic System for Mental Disorders in Children and Adolescents according to ICD-10 and DSM-IV, 2nd edition; SDQ-IH, Strengths and Difficulties Questionnaire, Inattention/Hyperactivity subscale (cut-off ≥ 7). **Assays:** ACS 180, Bayer Advia Centaur immunoassay system; CIA, competitive immunoassay; CLIA, ADVIA Centaur® chemiluminescent immunoassay; CLIMA, Immulite® 1000 chemiluminescent immunometric assay; CMIA, Abbott Architect® chemiluminescent microparticle immunoassay; ECLIA, electrochemiluminescence assay (Elecsys 2010); EnhCL, enhanced chemiluminescence; IFA, immunofluorescence assay; IRMA, immunoradiometric assay; MAB-FT4, monoclonal antibody-based competitive immunoassay for free T4; RIA, radioimmunoassay; TSH3-US, ADVIA Centaur® ultrasensitive TSH assay; US, ultrasensitive assay. **Medications:** ATX, atomoxetine; IR-MPH, immediate-release methylphenidate; OROS-MPH, osmotic release oral system methylphenidate. **Other:** FT3I, free T3 index; FT4I, free T4 index; IQR, interquartile range; Md, median; NR, not reported.

Year	First author	Population (% boys)	Diagnostic	Age	TT3 (nmol/L)	TT4 (nmol/L)	fT3 (pmol/L)	FT3I	fT4 (pmol/L)	FT4I	TSH (µUI/mL)
**1993**	Weiss RE	277 TDAH<br>(82.67%)	DSM-IIIR	9.8 ± 2<br>[4 ; 12]	NR<br>(RIA)	NR<br>(RIA)	—	—	—	8.4±1.7<br>(estimated)	2.3±1.6<br>(IRMA)
		106 Controls<br>(50.00%)		(matched)	—	—	—	—	—	—	2.5±1.5<br>(NR)
		46 Controls<br>(NR)		(matched)	—	—	—	—	—	8.5±1.5<br>(estimated)	—
**1994**	Elia J	53 TDAH<br>(100%)	DSM-III	9.16±1.65	2.47±.40<br>(N=51, RIA)	100.66±20.21<br>(N=40, IFA)	—	—	—	—	2.34±1.08<br>(N=53, IRMA)
		41 controls<br>(100%)		(matched)	2.48±.41<br>(N=42, RIA)	101.95±18.79<br>(N=41, IFA)	—	—	—	—	2.31±.85<br>(N=39, IRMA)
**1995**	Spencer T	132 TDAH<br>(78.79%)<br>including 106 with<br>pharmacological treatments	DSM-IIIR	11.4±3.33<br>[4 ; 18.8]<br>Md = 12	14/114 >3.0 (12.28%)<br>including<br>7/114 free-T3I (6.14%)<br>(NR)	< 12 years<br>96.9±17.5<br>≥ 12 years<br>86.9±15.6<br>(NR)	—	< 12 years<br>163±27.9<br>≥ 12 years<br>154±22.7	—	< 12 years<br>7.38±1.28<br>≥ 12 years<br>6.69±1.28	NR<br>1/126 >5mU/L<br>(US)
**1997**	Toren P	43 TDAH<br>(93.02%)<br>including 21 treated by	DSM-IIIR<br>& CATRS≥15	10.0±2.5<br>[6; 16]	2.36±.30<br>16/43 > 2.5<br>(37.21%)	101.6±16.3	—	—	17.2±2.4	—	1.33±.7

		methylphenidate			(EnhCL)	(EnhCL)			(MAB-FT4)		(US)
		28 controls<br>(96.43%)		11.2±3.1<br>[6; 16]	2.25±.30<br>6/28 > 2.5<br>(21.43%)	100.2±16.1	—	—	16.4±2.3	—	1.42±.7
**1997**	Valentine J	128 Predominantly<br>Hyperactive-Impulsive<br>(NR)<br>(86.72% treated by stimulants)	DSM-IV	[3; 20]	Md=2.3<br>SD=1.72<br>(ACS 180)	—	—	—	Md=13<br>SD=2.09<br>(ACS 180)	—	Md=2.25<br>SD= .85<br>(ACS 180)<br>One > 4.0
**1997**	Valentine J	48 Predominantly<br>Inattentive<br>(NR)<br>(91.67% treated by stimulants)	DSM-IV	[5; 17]	Md=2.3<br>SD=.35<br>(ACS 180)	—	—	—	Md=12<br>SD=1.54<br>(ACS 180)	—	Md=2.05<br>SD=1.00<br>(ACS 180)
**2003**	Stein MA	Overall 338 clinic-referred<br>patients (84.69%)	DSM-IV	9.2±3.18<br>[3; 18]	—	—	—	—	—	8.35±1.25<br>(RIA)	2.44±1.46<br>(IRMA)
		195 ADHD Combined<br>(NR)		9±3.1	—	—	—	—	—	8.45±.99	3.36±1.36
		54 ADHD Predominantly<br>Inattentive<br>(NR)		10.6±3.3	—	—	—	—	—	8.21±1.60	2.22±1.54
		84 other psychiatric disorders<br>(NR)		8.7±3.04	—	—	—	—	—	8.46±1.26	2.40±1.34
**2005**	Bereket A	72 ADHD<br>(NR)	DSM-IV	8.12±1.8<br>[6.47 ;<br>10.32]	2.75±.42<br>(Immulite)	145.45±21.88<br>(Immulite)	—	—	19.35±2.64<br>(Immulite)		2.86±1.89<br>(Immulite)
**2011**	Ercan ES	20 ADHD+CD<br>(95.00%)	DSM-IV	10.1±2.7<br>[6; 16]	—	—	6.16±.86<br>(N=19)<br>(NR)	—	15.58±1.93<br>(N=19)<br>(NR)	—	2.65±1.28<br>(N=19)<br>(NR)
**2013**	Ercan ES	35 ADHD<br>(57.14%)	DSM-IV	9.97±1.87	—	—	6.13±.83<br>(N=29)<br>(NR)	—	16.86±2.70<br>(N=30)<br>(NR)	—	2.62±1.21<br>(N=30)<br>(NR)
**2016**	Bala KA	34 ADHD<br>(67.65%)	DSM-5	7.68±3.20<br>(2-14)	—	—	—	—	18.79±2.01<br>(NR)	—	2.27±.76<br>(NR)
		27 healthy controls<br>(48.15%)		9.80±4.01<br>(3.5-17)	—	—	—	—	14.55±2.06	—	2.61±1.45
**2016**	Yüksek SK	89 ADHD	DSM-IV	9.41±1.45	—	—	7.33 ±1.06	—	16.22±2.06	—	2.15±1.14

		(83.1%)					[3.26; 9.97]<br>(Immulite)		[12.23;<br>21.63]<br>(Immulite)		
**2017**	Kuppili PP	30 ADHD<br>(93.33%)	DSM-IVTR	9.47 ± 2.43<br>[6; 15]	2.31±1.22<br>(CLIMA)	93.19±35.14<br>(CLIMA)	—	—	—	—	1.72±1.06<br>(CLIMA)
		30 Controls<br>(93.33%)		10.30 ± 2.79<br>[6; 16]	1.88±.54	120.23±29.35	—	—	—	—	2.14±1.02
**2018**	Gül A	11 ADHD<br>(81.8%)	DSM-IV	11.7±.67<br>[7; 15]	—	—	6.1±1.5<br>(CIA)	—	15.45±1.29<br>(CIA)	—	3.6±.4<br>(CIA)
**2018**	Langrock C	26 overweight ADHD<br>(65.39%)	DISYPS-II	8.7±1.8	—	—	6.91±.46<br>(CLIA)	—	15.45±1.29<br>(CLIA)	—	2.5±1.4<br>(CLIA)
		56 normal weight ADHD<br>(78.57%)		9.0±1.7	—	—	6.76±.62	—	16.73±1.29	—	2.2±1.0
		66 overweight<br>(42.42%)		10.0±1.5	—	—	6.91±.15	—	16.73±1.29	—	2.7±1.1
		82 normal weight<br>(62.20%)		9.1±1.7	—	—	6.61±.62	—	16.73±2.57	—	2.6±1.3
**2020**	Albrecht D<br>(Md and<br>IQR)	420 ADHD children<br>(80.00%)	SDQ-IH &<br>Clinician	10.6<br>(8.8 – 12.5)	—	—	6.1<br>(5.7 – 6.7)<br>(ECLIA)	—	18.1<br>(16.4 – 19.6)<br>(ECLIA)		2.1<br>(1.6 – 3.0)
		8265 Controls<br>(49.96%)	SDQ-IH &<br>Clinician	8.8<br>(6.1 – 11.5)			6.2<br>(5.7 – 6.7)		18.2<br>(16.8 – 19.8)		2.3<br>(1.7 – 3.0)
**2020**	Albrecht D<br>(Md and<br>IQR)	152 ADHD adolescents<br>(78.95%)	SDQ-IH	15.6<br>(14.4-16.7)	—	—	5.9<br>(5.4-6.7)	—	17.7<br>(16.2-19.4)		1.9<br>(1.3-2.7)
		2751 Controls<br>(46.24%)	SDQ-IH	15.65<br>(14.4-16.7)			5.7<br>(5.1 – 6.3)		18.1<br>(16.5-19.9)		1.8<br>(1.3-2.5)
**2020**	Kim WJ	64 Drug naïve ADHD<br>(64.06%)	DSM-IV	8.81±1.52<br>(b)<br>9.22±1.51<br>(g)	—	—	—	—	17.5±2.96<br>(CMIA)	—	2.91±1.66<br>(CMIA)
		29 MPH-treated ADHD<br>(79.49%)			—	—	—	—	19.18±2.19	—	2.11±1.18
		112 controls<br>(72.54%)		9.48±1.44<br>(b)<br>9.89±1.45<br>(g)	—	—	—	—	17.89±2.83	—	2.83±1.44

				9.20±1.65 (b) 9.36±1.44 (g)							
**2022**	Mei H	149 ADHD (62.42%)	DSM-5	8.9±2.78	1.77±.16	107.48±19.82	—	—	—	—	2.64±1.46
**2022**	Wang L-J	39 IR-MPH (76.92%)	DSM-5	9.0±2.3	1.97±.38 (CMIA)	109.03±20.85 (Siemens reagents)	—	—	13.77±2.57 (CMIA)	—	2.17±.95 (TSH3-US Advia Centaur)
		40 OROS-MPH (82.50%)		9.5±2.5	2.01±.31	110.57±21.37	—	—	13.26±1.29	—	2.26±1.03
		17 ATX (14 boys) (82.35%)		9.1±2.3	1.96±.33	104.52±20.60	—	—	12.87±1.29	—	1.94±.85
		22 None (72.73%)		8.8±1.9	1.89±.24	104.26±16.09	—	—	13.39±1.16	—	1.99±1.01
**2024**	Lukovac	67 ADHD (100%)	DSM-5	10.13 ± 1.41 [6.5; 12.5]	—	—	—	—	12.63±1.98 [9.38; 19]	—	2.73±1.18 [0.88; 6.8]
		66 controls (100%)		9.94 ± 1.52 [6.5; 12.5]	—	—	—	—	13.46±2.52 [9.2; 19.6]	—	2.60±1.08 [1.12; 6.98]
**2024**	Meng (Md and IQR)	4864 ADHD (81.44%)	DSM-5	7.24 ± 2.24	2.40 (2.17-2.68)	109.65 (96.43-121.08)	6.69 (6.15-7.27)	—	17.91 (16.50-19.47)	—	2.64 (1.91-3.58)
		4801 controls (57,72%)		6.34 ± 3.62	2.59 (2.30-2.94)	113.20 (98.36-125.80)	6.67 (6.12-7.24)	—	18.47 (16.86-20.10)	—	2.57 (1.86-3.51)

Methodological notes. Spencer et al. (1995) reported all values as mean ± SEM (Methods, p. 880: "Data are expressed as mean ± SEM unless otherwise stated"); standard deviations were reconstructed as SD = SEM × √n, using n = 132 for age and n ≈ 57 per subgroup for FT3I, T4, and FT4I stratified by age (the authors state that "half were aged 12 or older"). Spencer et al. (1995) did not report mean TT3 values but provided prevalence data: 14/114 ADHD patients (12.3%) had T3 above the upper laboratory limit of 195 ng/dL (3.0 nmol/L), and 7/114 (6.1%) had elevated FT3I; 1/126 (0.8%) had TSH above 5 mU/L. Of note, 106/132 ADHD patients (80.3%) had received prior psychopharmacological treatment, predominantly stimulants (55%), antidepressants (36%), and clonidine (24%), with no documented washout. Elia et al. (1994) reported only TT3, TT4, and TSH; available effective sample sizes per assay differed (TT3: n = 51/42; TT4: n = 40/41; TSH: n = 53/39 for ADHD/controls respectively). Where Spencer et al. (1995) reported FT3I elevation in the Elia et al. (1994) sample (4/53 ADHD, 3/41 controls), these figures were derived by Spencer's group applying their own laboratory cutoffs retrospectively and are not original to Elia et al. Meng et al. (2024) values correspond to the ADHD subgroup only (n = 4864), as extracted from Supplementary Table S2 (age and gender breakdown) and Table 3 (median and IQR for thyroid parameters) of the original publication.

Supplementary Table 3 — Thyroid parameters for boys with an abnormal TSH level at test time. Reference intervals shown are those reported by the referring laboratory at the time of testing; classification as abnormal was based on the laboratory's own thresholds, which occasionally differed from those printed on the report.

Age	ADHD Subtype	Free-T3	Free-T4	TSH	Comment
13.85	Combined	5.42<br>[3.54 ; 6.47]	13.27<br>[11.48 ; 22.70]	.436<br>[.55 ; 4.78]	Isolated low TSH
17.58	Combined	5.80<br>[3.10 ; 6.50]	20.30<br>[11.00 ; 22.00]	.287<br>[.400 ; 4.400]	Isolated low TSH
7.87	Combined	6.05<br>[5.10 ; 7.40]	16.20<br>[11.10 ; 18.10]	4.17<br>[.64 ; 4.16]	Borderline TSH<br>(just above upper limit)
17.19	Combined	6.20<br>[4.70 ; 7.20]	13.30<br>[10.7 ; 18.4]	5.591<br>[.510 ; 4.940]	Isolated high TSH
6.30	Inattentive	7.40<br>[3.10 ;6.50]	21.80<br>[10.30 ; 17.5]	4.533<br>[.400 ; 4.400]	Possible GRTH

Supplementary Table 4 — Thyroid parameters for girls with an abnormal TSH level at test time.

Age	ADHD Subtype	Free-T3	Free-T4	TSH	Comment
12.60	Combined	6.80<br>[3.10 ; 6.65]	15.10<br>[11.00 ; 22.00]	4.531<br>[.400 ; 4.400]	Elevated free-T3 and TSH
7.23	Inattentive	7.54<br>[3.53 ; 6.45]	17.70<br>[11.45 ; 22.65]	6.776<br>[.550 ; 4.780]	Elevated free-T3 and TSH
10.69	Combined	5.70<br>[3.10 ; 6.50]	17.70<br>[11.50 ; 22.70]	5.69<br>[.270 ; 4.20]	Isolated high TSH
12.68	Inattentive	4.98<br>[2.70 ; 6.40]	11.60<br>[10.00 ; 27.00]	5.86<br>[.200 ; 4.000]	Isolated high TSH
13.03	Combined	5.81<br>[4.70 ; 7.20]	15.00<br>[10.70 ; 18.40]	5.078<br>[.480 ; 4.170]	Isolated high TSH
13.22	Combined	5.90<br>[3.70 ; 7.00]	9.50<br>[6.17 ; 19.80]	3.945<br>[.400 ; 3.700]	Isolated high TSH
13.62	Inattentive	6.96<br>[3.07 ; 6.45]	12.90<br>[11.45 ; 22.65]	5.304<br>[.400 ; 4.400]	Elevated free-T3 and TSH
17.17	Combined	5.5<br>[3.50 ; 6.20]	11.50<br>[7.60 ; 13.30]	4.398<br>[.400 ; 4.400]	Isolated high TSH
7.84	Combined	6.30<br>[3.10 ; 6.80]	17.20<br>[12.00 ; 22.00]	7.41<br>[.27 ; 4.20]	Isolated high TSH
8.00	Inattentive	5.69<br>[4.10 ; 10.1]	10.04<br>[7.85 ; 15.96]	4.49<br>[.230 ; 4.000]	Isolated high TSH
8.85	Inattentive	6.30<br>[4.15 ; 7.99]	15.7<br>[11.97 ; 21.98]	5.54<br>[.27 ; 4.20]	Isolated high TSH

Supplementary Table 5 — Comparison of free-T3 medians (in pmol/L): Kapelari et al. (2008) vs. our subsample measured with ADVIA® Centaur/IECL.

Sex	Age group	Kapelari P50	Our P50	Difference	Difference (%)
Girls	6 – 10	6.20 (N=163)	6.40 (N=36)	+0.20	+3.23%
	11-14	5.90 (N=180)	6.03 (N=24)	+0.13	+2.20%
	15-18	5.30 (N=98)	5.20 (N=20)	-0.10	-1.89%
Boys	6 – 10	6.10 (N=219)	6.30 (N=157)	+0.20	+3.28%
	**11-14**	**6.00 (N=252)**	**6.47 (N=131)**	**+0.47**	**+7.83%**
	15-18	5.80 (N=211)	6.09 (N=36)	+0.29	+5.00%

Supplementary Table 6. Attrition of analytic samples across sequential regression models. The descriptive sample comprises 715 medication-naïve patients aged 6–17 years with a complete thyroid panel and normal TSH. Binary models contrast the 2IfT3 profile against an entirely normal thyroid profile; continuous models analyse the free-T3 to free-T4 ratio, which does not require profile classification and therefore retains all patients with a computable ratio.

	**Binary models (Table 3)**		**Continuous models (Table 4)**	
**Step**	**Excluded**	**Remaining**	**Excluded**	**Remaining**
Descriptive sample	—	715	—	715
Outcome computable	40	675	0	715
Immunoassay platform among the four main ones	29	646	32	683
**Model 1 — base model**		**646**		**683**
ODD status documented	90	556	96	587
**Model 2 — adding ODD**		**556**		**587**
CD status documented	91	465	99	488
**Model 3 — adding ODD and CD**		**465**		**488**

Outcome computable: for binary models, 36 patients presented an abnormal profile other than 2IfT3 and 4 lacked one or more laboratory reference limits; the ratio was computable for all 715 patients. Excluded and retained patients did not differ in age (11.7 vs 11.2 years, $p = 0.186$) or sex distribution ($\chi^2(1) = 1.59$, $p = 0.207$). All patients lost at the ODD and CD steps had undocumented status for the covariate added at that step. ODD, oppositional defiant disorder; CD, conduct disorder.

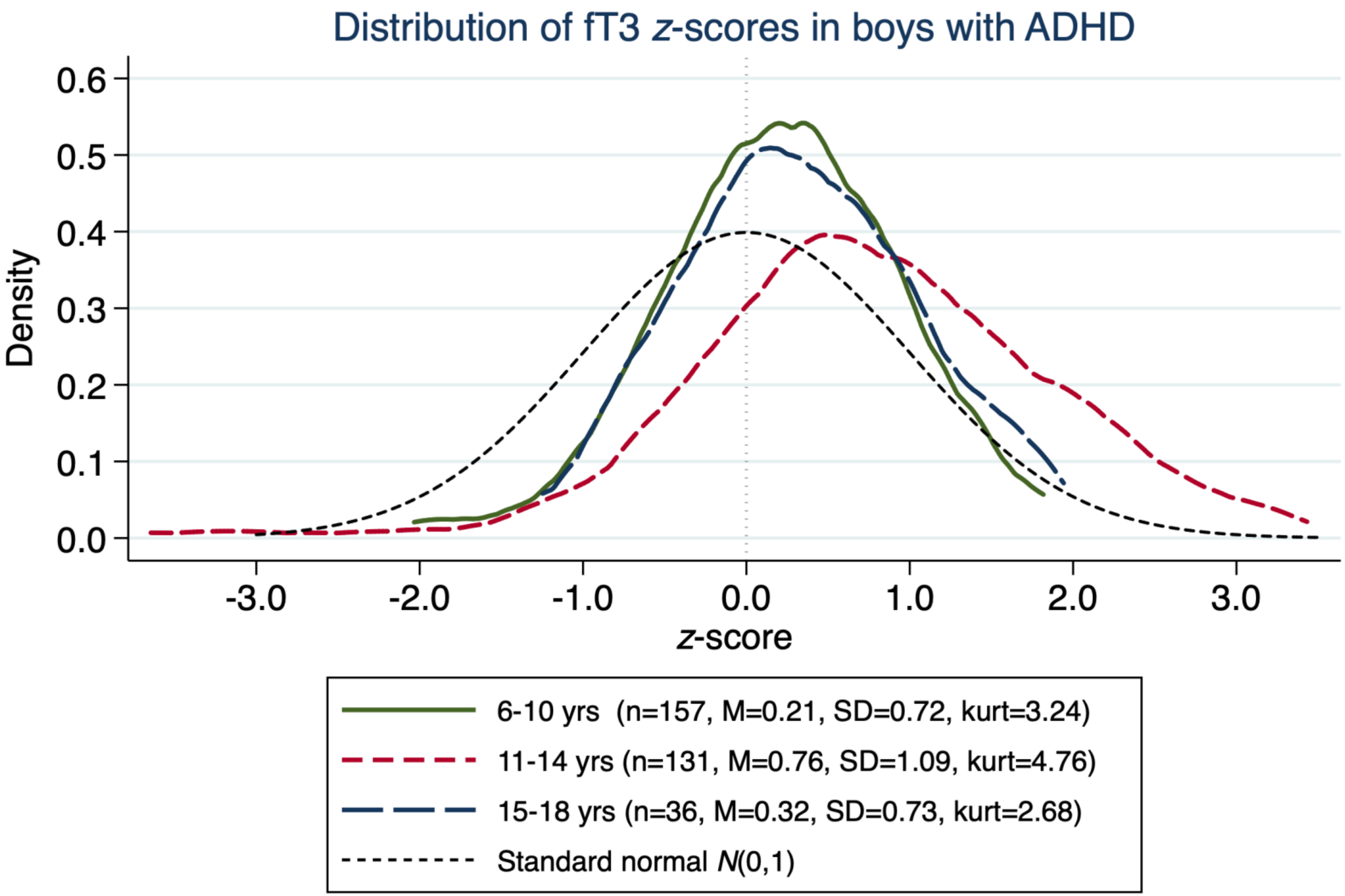

Pearson kurtosis (Gaussian reference = 3). z-scores computed against Kapelari et al. (2008) ADVIA Centaur pediatric reference values.

Supplementary Figure I — Kernel density distributions of free-T3 z-scores by age group in boys with ADHD (ADVIA Centaur platform, IECL technique; n=324). Z-scores were computed as z = (observed value − P50) / SD, with SD estimated as (P97.5 − P2.5) / 3.92 from Kapelari et al. (2008) sex- and age-specific reference values. The dashed line represents the standard normal distribution N(0,1). All three age groups show a positive shift relative to N(0,1). The 11–14 year group (red) exhibits the largest rightward displacement (mean z = +0.761) and heaviest right tail (kurtosis = 4.761; normal distribution kurtosis = 3), consistent with excess fT3 elevation concentrated in this age range. The 6–10 (green) and 15–18 year groups (blue) show more moderate shifts and near-normal kurtosis.

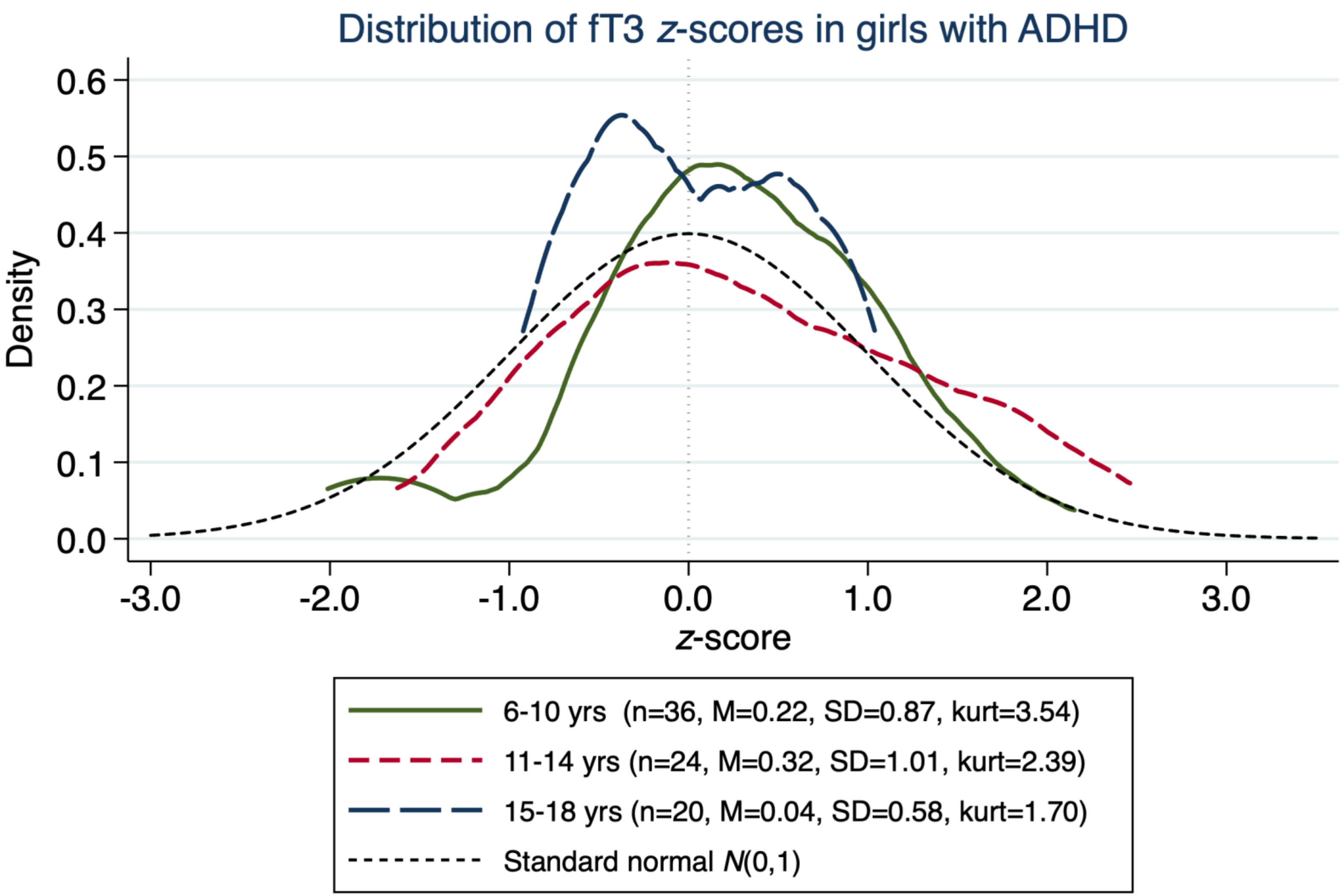

Pearson kurtosis (Gaussian reference = 3). z-scores computed against Kapelari et al. (2008) ADVIA Centaur pediatric reference values.

Supplementary Figure II — Kernel density distributions of free-T3 z-scores by age group in girls with ADHD (ADVIA Centaur platform, IECL technique; n=80). Z-scores were computed as z = (observed value − P50) / SD, with SD estimated under approximate normality assumption from the central 95% reference interval as (P97.5 − P2.5) / 3.92 from Kapelari et al. (2008) sex- and age-specific reference values. The dashed line represents the standard normal distribution N(0,1). In contrast to boys, the positive shift observed in the 6–10 year group (green; mean z = +0.219) persists in the 11–14 year group (red; mean z = +0.323) before near-complete normalization in the 15–18 year group (blue; mean z = +0.043), whose distribution closely approximates N(0,1). Kurtosis decreases progressively across age groups (3.540 → 2.389 → 1.695; normal distribution kurtosis = 3), consistent with a developmental trajectory toward normative thyroid function in adolescent girls with ADHD. This trajectory contrasts sharply with that observed in boys (Supplementary Figure I), where the 11–14 year group shows the largest fT3 elevation.

Supplementary Checklist. STROBE checklist for cross-sectional studies. Items are addressed with reference to the corresponding sections of the revised manuscript. ✅ indicates that the item is fully addressed; ⚠️ indicates a limitation inherent to the retrospective design, discussed explicitly in the manuscript.

	Item No	Recommendation and location in the manuscript
**Title and abstract**		
Title and abstract	1	(a) Indicate the study's design with a commonly used term in the title or the abstract ✅ "Retrospective observational study" appears explicitly in the title ("Isolated increased free-triiodothyronine in medication-naïve children and adolescents with ADHD: a retrospective observational study"). The Methods paragraph of the abstract repeats the term and states that the longitudinal component is an ancillary analysis subordinate to the primary cross-sectional design.<br>(b) Provide in the abstract an informative and balanced summary of what was done and what was found ✅ Structured abstract in four parts (Background, Methods, Results, Conclusion), following the format specified in the journal's author guidelines for original research articles. The Methods paragraph states explicitly that no non-ADHD comparison group was available, and the Conclusion states that the findings describe the frequency of a biochemical profile within an ADHD cohort rather than establishing an association with ADHD itself.
**Introduction**		
Background / rationale	2	Explain the scientific background and rationale for the investigation being reported ✅ §1: role of thyroid hormones in neurodevelopment, the need for age- and sex-specific pediatric reference intervals, brief summary of the PTSD and delinquency literatures with detail relegated to Supplementary Table 1, and a critical synthesis of prior ADHD-thyroid studies (Supplementary Table 2) identifying the methodological gap this study addresses.
Objectives	3	State specific objectives, including any prespecified hypotheses ✅ §1, final paragraph. Primary aim: to describe the frequency, within this cohort, of an isolated increase in free-T3 with normal free-T4 and TSH (the 2IfT3 profile), and how that frequency varies with age and sex. Secondary aim: to assess temporal stability in patients with repeat measurements. The pre-specified hypothesis (that profile frequency varies with age in a sex-dependent manner) is stated in §2.5.
**Methods**		
Study design	4	Present key elements of study design early in the paper ✅ "Retrospective observational study" stated in the title, the abstract, and §2.1, which also specifies that longitudinal analyses are ancillary to the primary cross-sectional design.
Setting	5	Describe the setting, locations, and relevant dates, including periods of recruitment, exposure, follow-up, and data collection ✅ University pediatric hospital outpatient unit, France, March 2004 – December 2022 (§2.1). Samples were analysed by community laboratories of the family's choosing (§2.2, §2.4).
Participants	6	(a) Give the eligibility criteria, and the sources and methods of selection of participants ✅ Inclusion: age 6–17 years, DSM-IV or DSM-5 ADHD diagnosis, medication-naïve, complete thyroid panel, normal TSH. Exclusion: incomplete or absent thyroid testing, abnormal TSH, pre-existing ADHD diagnosis under ongoing treatment (§2.2). Patients in whom no pharmacological treatment was indicated did not undergo testing and constitute the untested comparison group (§2.2, §3.1, Figure III).
Variables	7	Clearly define all outcomes, exposures, predictors, potential confounders, and effect modifiers. Give diagnostic criteria, if applicable ✅ Primary outcome: the 2IfT3 profile, defined as free-T3 above the upper reference limit with free-T4 and TSH both within their reference intervals (§2.2). Secondary outcomes: the continuous free-T3/free-T4 ratio and free-T3 z-scores (§2.5). Predictors: sex, age, ADHD presentation, ODD, CD. Analytical platform included as a covariate in all multivariable models (§2.4, §2.5).
Data sources / measurement	8*	For each variable of interest, give sources of data and details of methods of assessment. Describe comparability of assessment methods if there is more than one group ⚠️ §2.4 is devoted to this point. Complete assay documentation was available for 697 of 731 patients (95.3%), with the distribution across the four main platforms reported. Prior to the French Order of 26 November 1999, laboratories were not required to report immunoassay technique or reference intervals, and documentation remains incomplete for the earliest samples. Inter-platform heterogeneity was quantified (§3.6) and adjusted for in all multivariable models; LC-MS/MS standardization was unavailable for retrospective samples and is discussed as a limitation (§4.3, §5).
Bias	9	Describe any efforts to address potential sources of bias ✅ §4.2 (selection toward more severely symptomatic patients, single-clinician assessment blind to thyroid results, evolving documentation practices, unrecorded confounders) and §4.3 (untested biological parameters, immunoassay heterogeneity, reference-interval dependency). Comparison of excluded and retained patients on age and sex reported in §2.5.
Study size	10	Explain how the study size was arrived at ⚠️ No formal sample size calculation was performed. The study is retrospective and descriptive; sample size was determined by the number of eligible patients seen over the 19-year study period (§2.1, §3.1).
Quantitative variables	11	Explain how quantitative variables were handled in the analyses ✅ Thyroid values were handled both categorically (below, within, or above laboratory reference intervals) and continuously (free-T3/free-T4 ratio; free-T3 z-scores computed as z = (observed − P50)/SD with SD = (P97.5 −

		P2.5)/3.92). Age was analysed as a continuous variable in all regression models and grouped (6–10, 11–14, 15–18 years) only for the z-score comparison with published norms (§2.2, §2.5, §3.3).
Statistical methods	12	(a) Describe all statistical methods, including those used to control for confounding ✅ §2.5. Pearson chi-square for categorical comparisons; ANCOVA with partial-η²; Kolmogorov–Smirnov and Shapiro–Wilk tests for normality; logistic regression for the binary outcome; linear regression for the continuous ratio; all multivariable models adjusted for analytical platform. Significance threshold set at 0.05, two-sided. Software: Stata® version 15.1 (StataCorp LLC, College Station, TX, USA).<br>(b) Describe any methods used to examine subgroups and interactions ✅ The Sex × Age interaction was tested in every model, with sex-specific age effects reported separately, obtained for the binary models by linear combination of the age main effect and the interaction term (§2.5, §3.4, §3.6, Tables 3 and 4). Z-score analyses were stratified by sex and age group (§3.3).<br>(c) Explain how missing data were addressed ✅ Missing values were not imputed and no data were excluded on the basis of outlying values (§2.5). Attrition across models is documented step by step in §2.5 and Supplementary Table 6, with a comparison of excluded and retained patients on age and sex.<br>(d) If applicable, describe analytical methods taking account of sampling strategy ✅ Analytical platform was included as a covariate in all multivariable models (§2.4, §2.5). In the ancillary longitudinal analysis, standard errors were clustered on patient identifier to account for the non-independence of repeated measurements within individuals (§2.5, §3.5).<br>(e) Describe any sensitivity analyses ✅ Robustness was examined through three sequential models progressively adding ODD then CD diagnoses, for both the binary outcome (Table 3) and the continuous ratio (Table 4). Convergence across the three analytic approaches — binary classification, continuous ratio, and z-scores — is summarised in Table 5. No formal correction for multiple comparisons was applied, and the rationale for this choice is stated explicitly in §2.5.
**Results**		
Participants	13*	(a) Report numbers of individuals at each stage of study ✅ N = 2,042 consultations → N = 1,049 with blood tests → N = 795 aged 6–17 years with ADHD → N = 715 included after exclusion of incomplete panels (n = 64) and abnormal TSH (n = 16). Attrition across regression models (715 → 675 → 646 → 556 → 465 for binary models; 715 → 683 → 587 → 488 for continuous models) is reported in §2.5 and Supplementary Table 6 (§3.1, §3.4, Figure II).<br>(b) Give reasons for non-participation at each stage ✅ No blood test prescribed because no pharmacological treatment was indicated; incomplete thyroid panel; abnormal TSH; thyroid profile outside the binary contrast; infrequently used or undocumented analytical platform; undocumented comorbidity status (§2.5, §3.1, Supplementary Table 6).<br>(c) Consider use of a flow diagram ✅ Figures II and III present the breakdown of patients with and without blood testing.
Descriptive data	14*	(a) Give characteristics of study participants and information on exposures and potential confounders ✅ Table 1 (age at first consultation and at blood draws, by sex and age group) and Table 3, panel A (age, ADHD presentation, symptom counts, ODD and CD symptoms, by sex and thyroid profile). Platform distribution reported in §2.4 and §3.1.<br>(b) Indicate number of participants with missing data for each variable of interest ✅ Missing data are quantified for each variable that determines an analytic sample: analytical platform (34 of 731, 4.7%; §2.4), thyroid profile classification (40 of 715, 5.6%, with reasons; §2.5), ODD status (documented in 65% of eligible patients) and CD status (52%), with documentation completeness reported by study segment (§3.2, §5). Free-T3, free-T4 and TSH are complete by inclusion criterion.
Outcome data	15*	Report numbers of outcome events or summary measures ✅ Prevalence of the 2IfT3 profile and of all other thyroid profiles reported by sex in Table 2, with the numbers of patients in each profile category. Numbers of boys and girls with the 2IfT3 profile within each regression model are reported in Table 3, panel B.
Main results	16	(a) Give unadjusted estimates and, if applicable, confounder-adjusted estimates and their precision. Make clear which confounders were adjusted for and why they were included ✅ Unadjusted prevalence in Table 2; adjusted odds ratios with 95% confidence intervals and exact p-values in Table 3; adjusted regression coefficients with 95% confidence intervals and exact p-values in Table 4. All models are adjusted for analytical platform, the rationale being the documented inter-platform heterogeneity quantified in §3.6. Exact p-values are reported throughout rather than "not significant".<br>(b) Report category boundaries when continuous variables were categorized ✅ Reference-interval boundaries were those supplied by each reporting laboratory (§2.2, §2.4). Age groups for the z-score analysis (6–10, 11–14, 15–18 years) follow the strata of the published normative data (§3.3).<br>(c) If relevant, consider translating estimates of relative risk into absolute risk for a meaningful time period ✅ Not applicable: descriptive study without a concurrent comparison group. Predicted probabilities are nonetheless displayed on an absolute scale in Figures IV and VI.
Other analyses	17	Report other analyses done ✅ Z-score comparison with published pediatric norms in the largest immunoassay subgroup (§3.3); ancillary longitudinal analysis of temporal stability (§3.5); continuous free-T3/free-T4 ratio (§3.6); data for patients retested after age 18 reported for completeness (§3.5); convergence across the three analytic approaches summarised in Table 5.

**Discussion**		
Key results	18	Summarise key results with reference to study objectives ✅ §4, opening paragraph, and §6: approximately one third of patients presented the 2IfT3 profile; sex-specific developmental patterns; marked temporal instability; dissociation from ADHD presentation and comorbidities.
Limitations	19	Discuss limitations of the study, taking into account sources of potential bias or imprecision. Discuss both direction and magnitude of any potential bias ✅ The absence of a non-ADHD comparison group is stated as the first of three framing considerations in the opening paragraph of §4, developed in §4.1, and restated in §5 and §6. §5 synthesises all limitations: absent comparison group, retrospective single-centre design, marked sex imbalance, absent pubertal staging, partial documentation of comorbidities, unrecorded confounders, immunoassay heterogeneity with its magnitude quantified relative to physiological effects, and sparse unplanned longitudinal data.
Interpretation	20	Give a cautious overall interpretation of results considering objectives, limitations, multiplicity of analyses, results from similar studies, and other relevant evidence ✅ §4.4 and §4.5 present mechanistic accounts explicitly as hypothesis-generating. §4.6 opens with a statement that no forensic, psychopathy or trauma-exposure data were collected and that no such association was tested. The rationale for not applying a formal correction for multiplicity is stated in §2.5, together with the caveat that secondary findings should be read accordingly.
Generalisability	21	Discuss the generalisability (external validity) of the study results ✅ §5: single-centre design with testing prescribed on clinical judgment rather than by protocol, marked sex imbalance, and comorbidity-adjusted models resting on a selected subsample. §4.1 states that the absence of a comparison group precludes any claim of specificity to ADHD.
**Other information**		
Funding	22	Give the source of funding and the role of the funders for the present study ✅ No financial support was received for the research or for the publication of this article (Funding section). As no funding was received, no funder had any role in study design, data collection, analysis, interpretation, or manuscript preparation. Conflicts of interest are declared separately.

An Explanation and Elaboration article discusses each checklist item and gives methodological background and published examples of transparent reporting. The STROBE checklist is best used in conjunction with this article, freely available on the websites of PLoS Medicine, Annals of Internal Medicine and Epidemiology. Information on the STROBE Initiative is available at www.strobe-statement.org.
